# Prevalence, risk factors, and early prediction of cognitive impairment in patients with diabetes mellitus: a systematic review and meta-analysis

**DOI:** 10.3389/fendo.2026.1757367

**Published:** 2026-03-25

**Authors:** Qin Qin, Le Chen, Jingjing Wang, Luqing Zheng, Juan Zhao

**Affiliations:** 1 Department of Medical, Quzhou College of Technology, Quzhou, China; 2 School of Nursing, Xiangnan University, Chenzhou, China; 3 Institutes of Health Central Plains, Henan Medical University, Xinxiang Henan, China; 4 School of Nursing, Hunan Traditional Chinese Medical College, Zhuzhou, China; 5 Department of Psychiatry, First Hospital/First Clinical Medical College of Shanxi Medical University, Taiyuan, Shanxi, China

**Keywords:** cognitive impairment, diabetes mellitus, meta-analysis, predictive model, prevalence, risk factors, systematic review

## Abstract

**Introduction:**

Diabetes mellitus (DM) substantially increases the risk of cognitive impairment (CI). Currently, systematic evidence regarding its prevalence, risk factors, and early predictive models remains scarce, hindering the development of targeted preventive strategies. Thus, this meta-analysis aimed to comprehensively estimate the global CI prevalence in individuals with DM, identify associated risk factors, and assess the performance and potential application of early predictive models.

**Methods:**

Embase, Web of Science, the Cochrane Library, and PubMed were searched up to May 2025 to include observational studies reporting on the prevalence, risk factors, and predictive models of CI in individuals with DM. The Agency for Healthcare Research and Quality scale and the Newcastle-Ottawa Scale were employed to appraise the quality of included studies. Meta-analyses of prevalence, risk factors, and c-indices of predictive models were carried out using R 4.5.0. This study was registered in PROSPERO (CRD420250632808).

**Results:**

In total, 41 studies involving 18,768 patients with DM were included. After excluding three case–control studies, 38 studies were eligible for prevalence synthesis. Meta-analysis demonstrated an overall CI prevalence of 40.80% (95% CI: 33.91%–47.87%) among DM patients. Identified risk factors included demographic factors: age (odds ratio [OR]=2.18 95% confidence interval [CI]: 1.37–3.48), low income (OR = 2.25, 95%CI:1.28–3.96), and unmarried status (OR = 1.66, 95% CI:1.05–2.64); disease-related factors: Hemoglobin A1c (OR = 1.27, 95% CI:1.06–1.53), and hypoglycemia (OR = 1.87, 95% CI:1.30–2.71); and complications: diabetic nephropathy (OR = 1.64, 95% CI:1.19–2.25), diabetic retinopathy (OR = 1.71, 95% CI:1.27–2.42), depression (OR = 2.32, 95%CI: 2.32-5.33), and stroke (OR = 2.62, 95%CI:1.27–2.31). 8 studies constructed predictive models for CI, reporting a c-index of 0.83 (95%CI: 0.76–0.90) in the training sets and 0.81 (95%CI:0.74–0.89) in the validation sets.

**Conclusions:**

CI is highly prevalent among individuals with DM and is closely associated with multiple factors. These risk factors provide potential targets for early intervention. Although existing predictive models demonstrate encouraging performance, their clinical applicability remains limited owing to the small number of included studies and requires further validation. Future research should incorporate large, multicenter, and multiethnic cohorts and develop machine learning–based predictive models with broader applicability.

## Introduction

Diabetes mellitus (DM) is a common chronic non-communicable disease worldwide, with a rising incidence that increasingly threatens public health. According to the 11th edition of the International Diabetes Federation Diabetes Atlas, the global prevalence of DM reached 11.1% in 2024, affecting an estimated 589 million individuals, and is projected to rise to 12.2% by 2050, affecting about 853 million people (1). The growing global burden of DM, driven by population aging and lifestyle changes, places substantial economic and social pressures on individuals, families, and healthcare systems (2, 3). Beyond traditional concerns regarding glycemic control and macrovascular or microvascular complications, growing attention has been directed toward DM-related cognitive impairment (CI), a complication with profound functional and prognostic implications. Emerging evidence demonstrates that individuals with DM exhibit a markedly elevated risk of CI in comparison with the general population (4, 5).

The pathophysiology of CI in DM patients is multifactorial, involving chronic hyperglycemia, oxidative stress, neuroinflammation, cerebral microvascular damage, insulin resistance, and altered brain metabolism, all of which contribute to accelerated neurodegeneration (6, 7). CI in DM patients often manifests as deficits in attention, executive function, memory, and processing speed, substantially impairing patients’ daily living and social functioning. Current studies have demonstrated that CI significantly impairs quality of life and elevates the risk of falls, accidents, and unplanned hospital readmissions, thereby complicating long-term disease management (8, 9). As cognitive decline progresses, patients’ medication adherence and self-care capacity often deteriorate, leading to poor glycemic control, higher risk of complications, and escalating healthcare costs, ultimately placing considerable psychological and financial burdens on families and society (10). Importantly, cognitive dysfunction in DM has also been associated with increased mortality and greater healthcare utilization, suggesting its clinical and public health significance (7).

Epidemiological evidence demonstrates that individuals with DM have a 1.5 to 2.5-fold elevated risk of dementia in comparison to those without DM (11). Moreover, MCI represents a critical “window of opportunity” for preventing or delaying dementia. A 20-year retrospective study reported that about 32% of individuals with MCI progressed to dementia within a median follow-up of two years (12). Given this progression risk, early identification and intervention for CI among individuals with DM are crucial. However, despite growing research interest in this topic, the current evidence base remains fragmented and lacks integration. Failure to recognize CI at an early stage may result in suboptimal glycemic control, inappropriate therapeutic decisions, and delayed implementation of preventive strategies, thereby increasing long-term morbidity and healthcare costs.

To date, several systematic reviews have focused separately on either the prevalence (5) or risk factors (13) of MCI in patients with type 2 diabetes mellitus (T2DM). A few recent reviews have evaluated early prediction models (14), reporting moderate-to-high predictive accuracy but also highlighting issues such as small sample sizes, lack of external validation, and methodological bias. Consequently, the current evidence remains fragmented. Systematic reviews and meta-analyses are lacking, which may hinder the development of standardized screening strategies, impede risk stratification efforts, and limit translation of prediction models into routine clinical practice. The inconsistent reporting of prevalence, risk factors, and model performance reduces comparability across studies and complicates future model development and validation. To address these gaps, the present systematic review and meta-analysis was designed to answer the following research questions: (i) What is the pooled prevalence of CI (including MCI) among individuals with DM? (ii) What are the major risk factors for CI in this population? (iii) What prediction models have been developed for CI/MCI in DM, and how do they perform (e.g., AUC)?

Therefore, this systematic review and meta-analysis aimed to provide updated pooled prevalence estimates of CI across diverse DM populations, synthesize both well-established and emerging risk factors, and critically assess the characteristics, performance metrics (e.g., AUC), and applicability of early prediction models for CI/MCI in individuals with DM. By integrating epidemiological burden, etiological factors, and metrics of predictive models, this study seeks to provide references for risk stratification, early screening, and intervention strategies for DM.

## Methods

### Study registration

The study protocol was registered in PROSPERO (CRD420250632808). This meta-analysis was carried out in accordance with the Preferred Reporting Items for Systematic Reviews and Meta-Analyses (PRISMA) guidelines (15).

### Eligibility criteria

The inclusion criteria were as follows: (i) studies enrolling adult patients diagnosed with DM, including type 1, type 2, or mixed populations; (ii) studies reporting data on the prevalence, risk factors, or predictive models of CI. CI was defined as mild CI (MCI) or broader CI diagnosed using validated clinical criteria (e.g., DSM or ICD) or standardized cognitive assessment tools (e.g., MMSE, MoCA, or equivalent validated instruments with predefined cut-off values); (iii) observational study designs, including cohort, cross-sectional, or case–control studies; (iv) full-text articles published in English in peer-reviewed journals.

Exclusion criteria were as follows: (i) editorials, reviews, conference abstracts, dissertations, or laboratory-based experimental studies; (ii) studies not reporting cognitive outcomes or lacking sufficient data for analysis; (iii) studies on individuals with comorbid neurological or psychiatric conditions known to independently affect cognitive function (e.g., stroke, Parkinson’s disease, severe psychiatric disorders such as schizophrenia or bipolar disorder, active malignancy, or advanced frailty defined as Clinical Frailty Scale ≥ 6), which may limit the generalizability to the broader population with DM.

### Data sources and search strategy

Four electronic databases (PubMed, Embase, Web of Science, and the Cochrane Library) were systematically searched from inception to May 2025. The search strategy combined Medical Subject Headings (MeSH) and free-text terms related to DM, CI, machine learning, risk factors, and predictors. The strategy was adapted to the specific features of each database (**Supplementary Table 1**). Only studies published in English were included to ensure consistency in terminology and reporting standards. Gray literature, such as conference proceedings, dissertations, and non-peer-reviewed reports, was not included, as these sources typically lack sufficient methodological detail and quality assessment, which may compromise the validity and reproducibility of the review.

### Literature selection and data extraction

To ensure methodological rigor and reproducibility of the findings, a standardized literature screening process was implemented. All retrieved records were imported into EndNote, and duplicates were excluded automatically. Two reviewers (QQ and LC) independently screened titles and abstracts to exclude articles irrelevant to the research question. For studies that potentially met the inclusion criteria, the full text was reviewed to determine eligibility according to the predefined inclusion and exclusion criteria. Any disagreements during the screening process were addressed through discussion. Two reviewers independently screened the studies, achieving excellent inter-rater agreement (Kappa = 0.935). Any disagreements were resolved by consulting a third reviewer (JZ).

The following data were independently extracted by two reviewers (QQ and JJW): publication year, country, first author, study design, diagnostic tool for CI, participant age, sex, number of CI cases, number of DM cases, risk factors, and type of predictive model used. Extracted data were cross-checked to ensure accuracy and consistency. Any disagreements were addressed by consulting a third reviewer (JZ) to confirm the final data.

### Literature quality assessment

The 11-item checklist recommended by the Agency for Healthcare Research and Quality (AHRQ) was utilized to appraise the quality of cross-sectional studies. Each item of this scale was rated as “Yes,” “No,” or “Unclear” (16). The Newcastle-Ottawa Scale (NOS) was applied to appraise the quality of case-control and cohort studies. The scale comprised 9 points and evaluated studies based on the selection of participants, comparability between groups, and ascertainment of exposure or outcome. Studies were assessed as high quality (7–9 points), moderate quality (5–6 points), or low quality (<5 points) (17). Two investigators independently performed the quality assessment, and any disagreements were addressed through discussion or adjudication by a third investigator.

### Certainty of evidence assessment

The certainty of evidence for pooled associations of risk factors was assessed using the Grading of Recommendations Assessment, Development and Evaluation (GRADE) approach (18). As all included studies were observational, the certainty of evidence was initially low. Evidence was downgraded, where appropriate, for inconsistency (substantial heterogeneity), imprecision (wide or null-crossing confidence intervals), and a limited number of studies. No upgrading was applied.

### Synthesis methods and analysis

The prevalence, risk factors, and c-index values of CI were pooled in the meta-analysis. Subgroup analysis for the prevalence was performed based on diagnostic methods, region, and demographic variables. A *P* value < 0.05 was considered statistically significant. Publication bias was examined using funnel plots and Egger’s linear regression test. Risk factors were extracted from multivariable analyses reported in individual studies. For studies that did not provide standard errors or confidence intervals (CIs) for the c-index, these values were estimated using the formula proposed by Debray TP et al. (19). Heterogeneity was quantified by the I² statistic. An I² > 50% suggested substantial heterogeneity, in which case a random-effects model was applied. Otherwise, a fixed-effects model was employed for meta-analysis. Prediction model studies were analyzed separately, focusing on model characteristics and performance metrics. No statistical synthesis was performed across these study categories, as their research objectives and outcome measures differed fundamentally. All statistical analyses were performed using R version 4.5.0 (R Foundation for Statistical Computing, Vienna, Austria).

## Result

### Study selection

**Figure 1:**
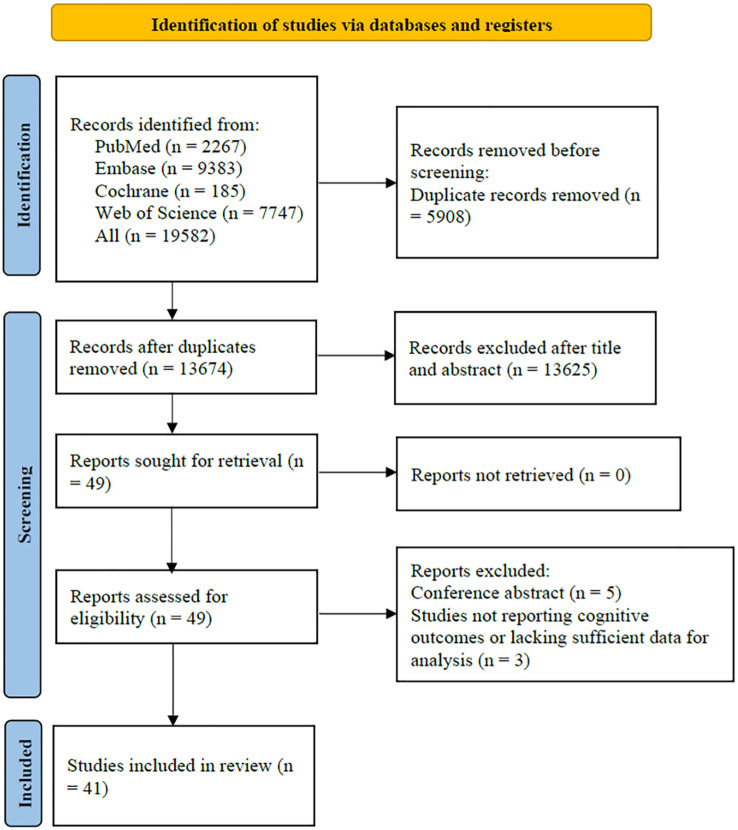
PRISMA flow diagram illustrating the literature screening process.

There were 19,582 articles retrieved from the databases. After removing 5,908 duplicates, the titles and abstracts of 13,674 studies were screened, and 13,625 irrelevant studies were excluded due to inappropriate study design or unrelated topics. The full texts of 49 articles were assessed for eligibility. Among them, 5 were excluded because they were unpublished conference abstracts, and 3 studies were further excluded for not reporting cognitive outcomes or lacking sufficient data for analysis. Ultimately, 41 studies were included in the meta-analysis (20–60) (**Figure 1**).

### Study characteristics

The included studies were published between 1 January 2008 and 31 May 2025, covering 14 countries worldwide across six continents (Asia, Europe, North America, South America, Oceania, and Africa). These studies were conducted in China (21, 24, 28, 29, 31–33, 36, 40, 41, 43–46, 49, 51–53, 56, 57), India (42, 58–60), Ethiopia (47, 50), Saudi Arabia (30, 34), the Philippines (26, 48), Australia (20), the UK (23), Japan (25), Cameroon (27), Malaysia (35), Chile (37), Thailand (38), Morocco (39), and Mexico (22). There were 18,768 DM patients enrolled. All 41 studies reported the number of individuals with DM and CI, totaling 6200 cases, with a mean age ranging from 50 to 80 years. Regarding the diagnostic criteria for CI, 13 studies employed the MMSE (20, 25, 27, 29, 39, 43, 47, 50, 53, 55, 56, 59, 60), 20 studies utilized the MoCA (21, 26, 30–32, 34–36, 41, 42, 44–46, 49, 51, 52, 54, 57), and 8 studies applied other diagnostic methods (22–24, 28, 33, 37, 38, 48), such as the diagnostic and statistical manual of mental disorders, 3rd ed., revised (DSM-III-R) (61). 34 studies adjusted for potential confounders in risk factors analyses, including demographic variables (sex, age, education level), lifestyle habits (exercise, smoking, alcohol use), disease-related factors (DM duration, body mass index [BMI], depression), comorbidities (diabetic nephropathy, diabetic retinopathy, hypertension, hypoglycemia, stroke), and metabolic markers (fasting plasma glucose [FPG], hemoglobin A1c [HbA1c], homocysteine [HCY], high-density lipoprotein [HDL]) (20, 23, 24, 26, 28–40, 43, 45–60). Additionally, 8 studies developed predictive models for CI (40, 41, 44, 45, 49, 51, 55, 57), including nomograms based on logistic regression and machine-learning methods such as CHAID decision trees and random vector functional link networks (**Supplementary Table 2**).

### Quality assessment

Among these included studies, 36 were cross-sectional (21–32, 34–38, 40–42, 44–54, 56–60), 3 were case-control (33, 43, 55), and 2 were cohort studies (20, 39). The quality of cross-sectional studies was appraised using the AHRQ scale: 7 studies (19.4%) were of high quality (≥ 8 points), and the remaining 29 studies (80.6%) were of moderate quality (5–7 points). Low scores were particularly noted in the items “indicate if evaluators of subjective components of study were masked to other aspects of the participants” and “describe any assessments undertaken for quality assurance purposes,” indicating that most studies inadequately reported measures to control subjective bias and ensure quality. The quality of case-control studies was appraised by the NOS scale. Their total scores ranged from 7 to 8, all classified as high quality. Most studies scored lower on the “non-response rate” item, indicating that the response rates for case and control groups were often not reported. Cohort studies were also assessed using the NOS scale. Their total scores ranged from 7 to 8, all classified as high quality. Scores were lower on the “adequacy of follow-up of cohorts” item, suggesting that some studies had insufficient follow-up duration or incomplete reporting of losses to follow-up. Nevertheless, all included studies met the minimum quality standards, providing a reliable basis for subsequent analyses (**Supplementary Tables S3, S4**).

### Quantitative synthesis of prevalence

#### Pooled prevalence of cognitive impairment

**Figure 2:**
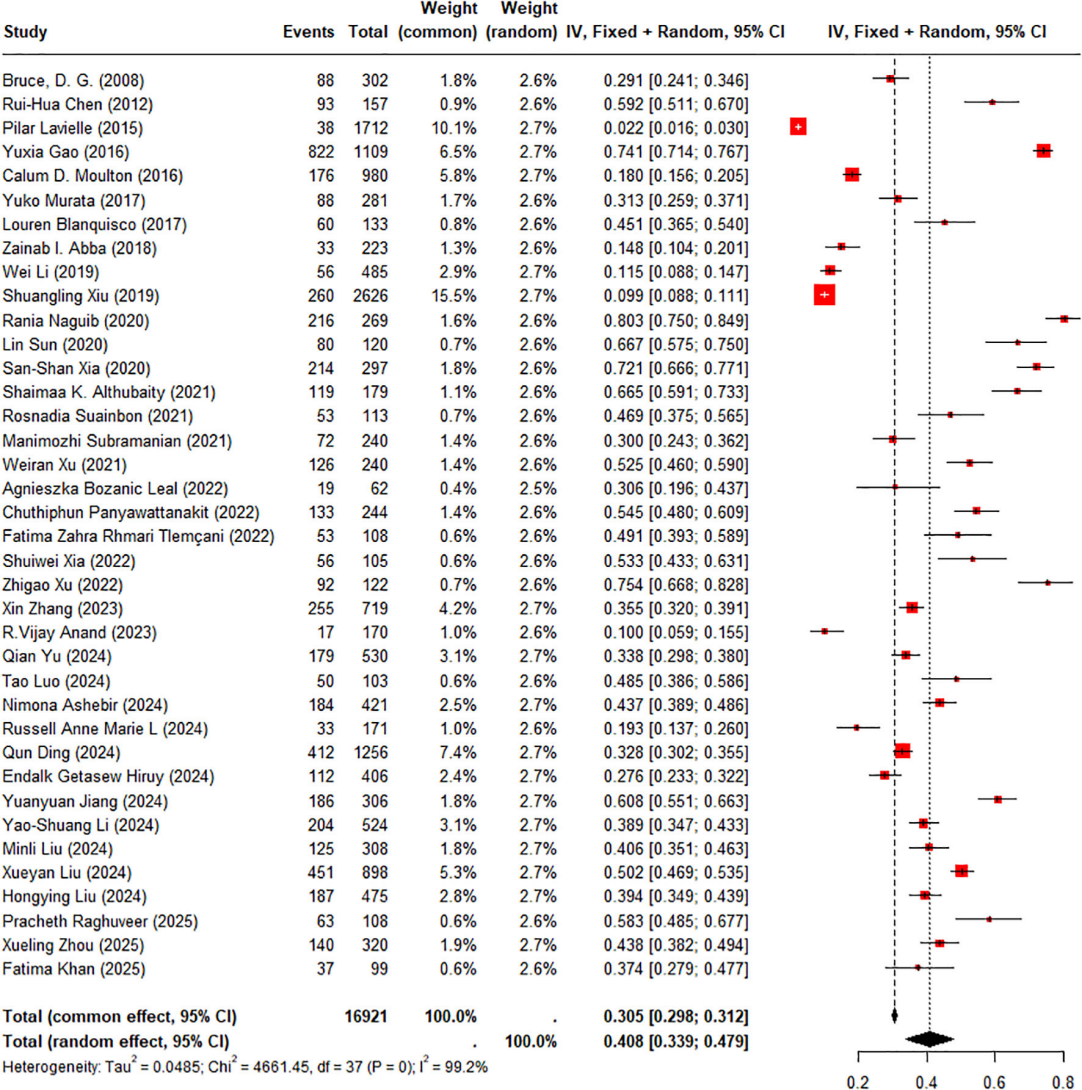
Forest plot of pooled prevalence of cognitive impairment in DM.

The prevalence estimates of CI were derived from 36 cross-sectional studies and two cohort studies that reported baseline data. The three case-control studies were not included in the pooled analysis of prevalence, as their design did not allow estimation of population-level prevalence. Across these 38 studies, a total of 16921 individuals with DM were enrolled, among whom 5582 were diagnosed with cognitive disorders (including CI, MCI, and dementia). A random-effects model was used for meta-analysis. The pooled prevalence of overall cognitive disorders was 40.80% (95% CI: 33.91%–47.87%) (**Figure 2**), with significant heterogeneity (I² = 99.2%). Given that MCI, broader CI, and dementia were distinct clinical entities, subgroup analysis by disease type was performed. The pooled prevalence was 33.66% (95% CI: 25.57%–42.26%) for broader CI, 46.85% (95% CI: 38.15%–55.66%) for MCI, and 13.73% (95% CI: 6.53%–23.02%) for dementia. These findings indicated substantial variations across diagnostic stages.

#### Subgroup analysis

Subgroup analysis stratified by diagnostic criteria for CI demonstrated that the CI prevalence in DM patients was 51.31% (95% CI: 43.36%–59.22%) in the MoCA group, higher than 33.78% (95% CI: 27.71%–40.13%) in the MMSE group and 24.66% (95% CI: 9.68%–43.70%) in the group using other diagnostic tools (**Supplementary Figure 1**). When stratified by region, the CI prevalence in DM patients was 45.31%(95% CI:37.91%- 52.81%) in Asia and 32.83% (95% CI:18.28%-49.28%) in Africa (**Supplementary Figure 2**). However, except for Asia and Africa, most continental subgroups were based on a single study. Therefore, these regional estimates should be interpreted with caution. When stratified by demographic variables, the prevalence of CI in DM patients was 44.50% (95% CI: 35.82%–53.36%) in females, 62.32% (95% CI: 35.93%–85.36%) in patients aged > 60 years, 44.75% (95% CI: 32.73%–57.08%) in unmarried or divorced individuals, 54.29% (95% CI: 39.88%–68.35%) in patients with ≤ 12 years of education, 44.89% (95% CI: 25.94%–64.62%) in rural residents, 52.14% (95% CI: 35.13%–68.91%) in unemployed patients, 49.71% (95% CI: 31.24%–68.23%) in patients lacking regular exercise, 44.05% (95% CI: 34.70%–53.61%) in smokers, and 43.33% (95% CI: 34.35%–52.47%) in alcohol consumers (**Table 1**).

**Table 1:** Subgroup analysis of the prevalence of CI in DM patients.

Subgroup	Classification	Number of studies	Number of events	Number of observations	Prevalence (95%CI) (%)	I^2^ (%)
Diagnostic category	Cognitive disorders (All)	38	5582	16921	40.80 (33.91-47.87)	99.2
	Broader cognitive impairment (CI)	23	2907	11418	33.66 (25.57-42.26)	99.1
	Mild cognitive impairment (MCI)	15	2420	5503	46.85 (38.15-55.66)	98.0
	Dementia	5	262	2111	13.73 (6.53-23.02)	96.2
Diagnosis Tool	MoCA	20	3066	6669	51.31 (43.36-59.22)	97.2
	MMSE	10	979	2863	33.78 (27.71-40.13)	90.8
	Others	8	1537	7389	24.66 (9.68-43.70)	99.7
Continent	Asia	30	4879	12707	45.31 (37.91-52.81)	99
	Africa	4	382	1158	32.83 (18.28-49.28)	96.2
	Europe	1	179	980	17.96 (15.62-20.43)	–
	North America	1	38	1712	2.22 (1.57-2.98)	–
	South America	1	19	62	30.65 (19.71-42.76)	–
	Oceania	1	88	302	29.14 (24.14-34.40)	–
Variables						
Sex	Female	32	2673	8343	44.54 (35.59-53.67)	98.9
	Male	32	2055	7034	35.52 (27.84-43.58)	98.5
Age	≤60 years	4	210	564	38.62 (13.54-67.42)	98.1
	>60 years	4	253	459	62.32 (35.93-85.36)	97.1
Marital status	yes	9	1432	5146	36.95 (25.74-48.91)	99.1
	others	9	512	1493	44.75 (32.73-57.08)	96.5
Education	≤12 years	10	1224	2390	54.29 (39.88-68.35)	97.6
	>12 years	10	273	858	33.92 (21.48-47.58)	94.4
Residence	Urban	7	674	3672	27.71 (19.17-37.15)	98.2
	Rural	7	299	944	44.89 (25.94-64.62)	97.1
Employment	yes	6	168	373	38.32 (18.76-60.00)	93.7
	no	6	533	1058	52.14 (35.13-68.91)	96.8
Exercise	Yes	11	764	2870	31.09 (19.28-44.28)	97.5
	No	11	1058	3630	49.71 (31.24-68.23)	99.4
Smoking	Yes	21	1134	2598	44.05 (34.70-53.61)	96.1
	No	21	2494	7181	42.62 (34.13-51.34)	98.9
Alcohol	Yes	18	1078	2645	43.33 (34.35-52.47)	99.1
	No	18	2671	7626	38.19 (28.87-47.96)	96.6

CI, Cognitive impairment; DM, Diabetes mellitus; MCI, Mild cognitive impairment.

#### Publication bias

**Figure 3:**
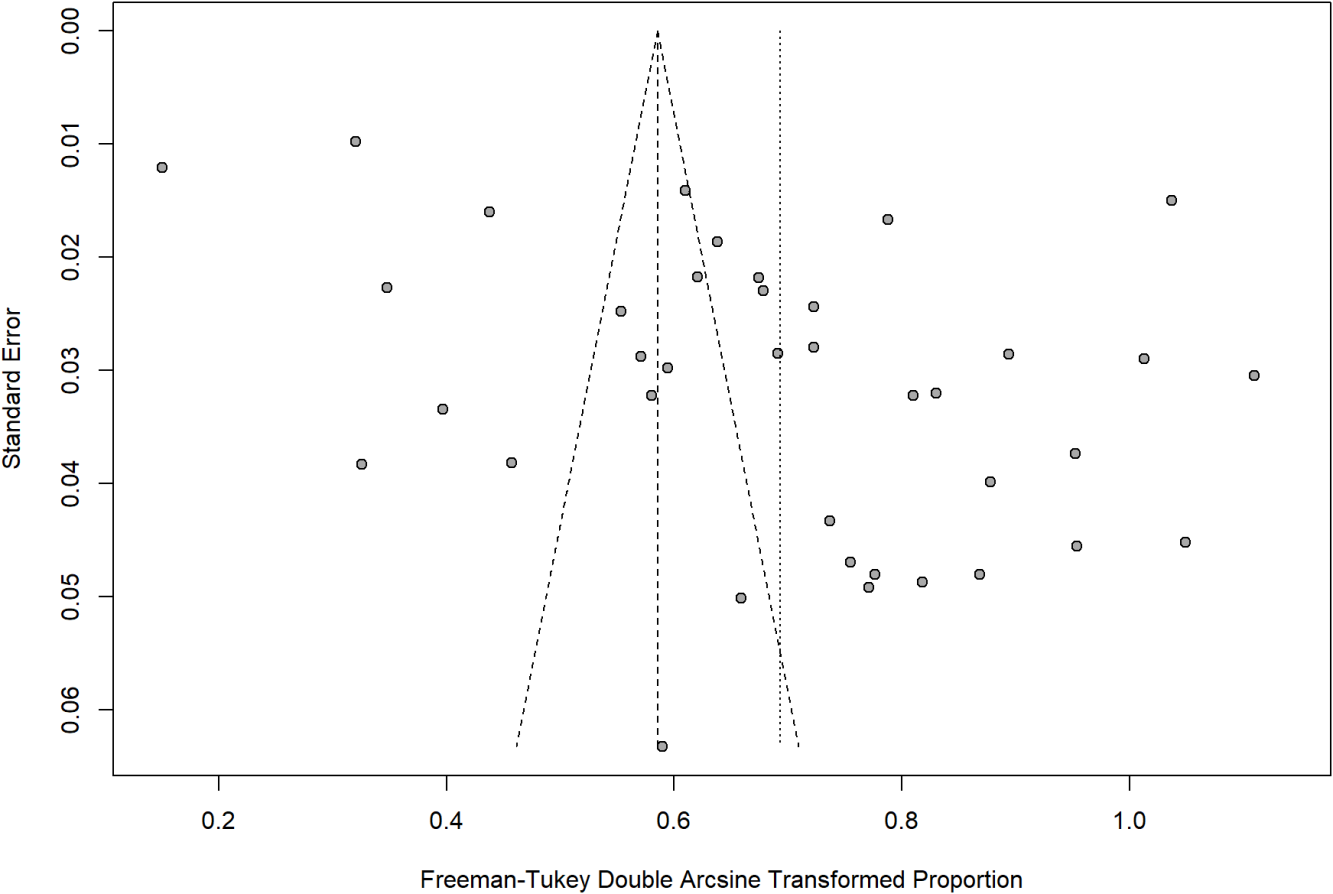
Funnel plot of the prevalence of cognitive impairment in DM.

Publication bias was assessed for the prevalence when more than 10 studies were available (62). The funnel plot was asymmetrical (**Figure 3**). Egger’s regression test indicated significant small-study effects (P = 0.0023). After applying the trim-and-fill method, 17 studies were imputed. The adjusted pooled prevalence was 22.23% (95% CI: 14.79–30.67%), lower than the original estimate of 40.80% (95% CI: 33.91–47.87%). This substantial reduction suggested that small-study effects or potential publication bias may have influenced the magnitude of the pooled estimate. Moreover, given the considerable between-study heterogeneity (I² = 99.2%), these findings should be interpreted with caution.

### Quantitative synthesis of risk factors

Risk factors reported in at least two studies were quantitatively synthesized using pooled odds ratios (ORs). The following variables were significantly associated with increased risk of CI among individuals with DM demonstrated that age > 60 years (OR = 2.18, 95% CI: 1.37–3.48), low income (OR = 2.25, 95% CI: 1.28–3.96), unmarried status (OR = 1.66, 95% CI: 1.05–2.64), Depressionm (OR = 2.32, 95%CI: 1.01-5.33), HbA1c (OR = 1.27, 95% CI: 1.06–1.53), HCY (OR = 1.64, 95%CI:1.25-2.15), hypoglycemia (OR = 1.87, 95% CI: 1.30–2.71), diabetic nephropathy (OR = 1.64, 95% CI: 1.19–2.25), diabetic retinopathy (OR = 1.68, 95% CI: 1.17–2.42), and history of stroke (OR = 2.62, 95% CI: 1.05–6.56) were major risk factors for the onset of CI in DM (**Table 2**; **Supplementary Figure S4-S30**).

**Table 2:** Synthesized effect size of risk factors for cognitive impairment in DM patients.

Factors	Value	Number of studies	OR (95%CI)	I^2^ c(%)	Grade
Age	Per 1-year increase	13	1.28 (1.01-1.62)	98.5	Very low
	Older (>60years)	11	2.18 (1.37-3.48)	86.6	Very low
Sex	Female	15	1.12 (0.85-1.49)	57.5	Low
Living area	Urban	5	0.42 (0.19-0.94)	81.5	Very low
Living alone (*vs* living with family)	Family	3	0.23 (0.07-0.75)	81.4	Very low
Income	Low income	5	2.25 (1.28-3.96)	62.0	Low
Marital status	Unmarried	4	1.64 (1.24-2.17)	9.0	Moderate
	Widowed	2	2.18 (1.27-3.74)	0.0	Very low
	Single	1	2.20 (0.91-5.33)	–	Very low
	Divorce	1	2.50 (0.97-6.46)	–	Very low
Education	Per 1-year increase	7	0.84 (0.81-0.87)	14.2	Moderate
Employment	Employed	5	0.71 (0.48-1.03)	0.0	Low
Smoking	yes	6	1.58 (0.88-2.81)	89.1	Very low
Alcohol	yes	7	1.56 (0.90-2.70)	67.6	Low
BMI	Per 1 kg/m² increase	2	0.95 (0.90-1.01)	39.1	Very low
Waist circumference	Per 1-cm increase	4	0.97 (0.70-1.33)	80.4	Very low
Exercise	yes	3	0.41 (0.16-1.08)	81.9	Very low
Comorbidities	yes	2	2.23 (0.80-6.21)	60.6	Very low
Duration diabetes	Per 1-year increase	9	1.41 (0.96-2.07)	91.1	Very low
	≥10 years	4	1.26 (0.96-1.71)	0.0	Low
Depression	yes	6	2.32 (1.01-5.33)	82.6	Very low
Diabetic nephropathy	yes	3	1.64 (1.19-2.25)	0.0	Low
Diabetic retinopathy	yes	2	1.68 (1.17-2.42)	0.0	Very low
Hypertension	yes	6	0.56 (0.25-1.28)	82.1	Very low
Hypoglycemia	yes	5	1.87 (1.30-2.71)	44.4	Low
Stroke	yes	3	2.62 (1.05-6.56)	83.6	Very low
FPG	Per 1 mmol/L increase	2	2.68 (0.51-14.14)	96.5	Very low
HbA1c	Per 1% increase	8	1.27 (1.06-1.53)	82.2	Very low
HCY	Per 1 μmol/L increase	2	1.64 (1.25-2.15)	0.0	Very low
HDL	Per 1 mmol/L increase	2	0.97 (0.93-1.02)	4.0	Very low
HOMA-IR	Per 1-unit increase	2	1.79 (0.49-6.57)	74.0	Very low
TC	Per 1 mmol/L increase	2	1.08 (0.88-1.32)	0.0	Very low

For continuous variables, pooled odds ratios represent the effect per one-unit increase in the corresponding measurement. BMI, Body mass index; FPG, Fasting plasma glucose; HbA1c, Hemoglobin A1c; HCY, Homocysteine; HDL, High-density lipoprotein; HOMA-IR, Homeostatic Model Assessment of Insulin Resistance; TC, Total cholesterol.

According to the GRADE assessment, the certainty of evidence for most risk factor associations ranged from low to very low. The certainty evidence was moderate for higher educational attainment and unmarried status, reflecting relatively consistent and precise effect estimates. The certainty evidence for several factors, including low income, hypoglycemia, employment status, and duration of DM ≥ 10 years, was low. However, the certainty of evidence for the majority of associations was downgraded to very low due to substantial heterogeneity, imprecision, or limited numbers of studies.

### Performance of prediction models

**Figure 4:**
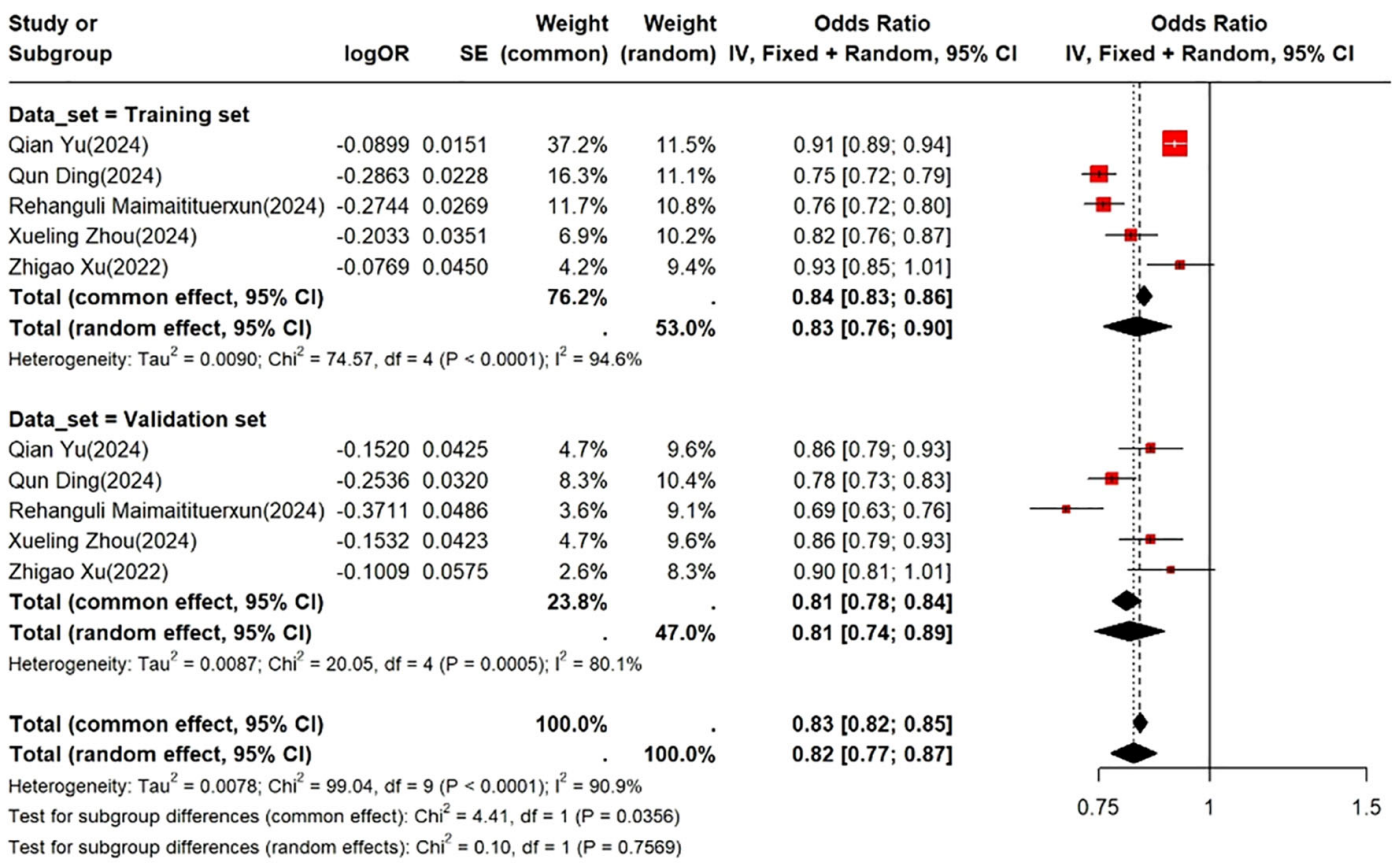
Forest plot of C-index as a risk factor.

Eight studies developed prediction models for CI in individuals with DM. Due to substantial methodological heterogeneity in model construction, predictor selection, outcome definitions, and validation strategies, model characteristics were summarized descriptively. In addition, to provide an overall estimate of model discrimination, reported c-index (AUC) values were quantitatively pooled using a random-effects model. The pooled c-index was 0.83 (95% CI: 0.76–0.90) in the training sets and 0.81 (95% CI: 0.74–0.89) in the validation sets (**Figure 4**). Further details regarding model type, CI case definitions, validation methods, sample sizes, and AUC values are presented in **Table 3**.

**Table 3:** Predictive models for cognitive impairment in DM patients.

Study	Type of model	DM(CI)	Validation method	Training/validation sample size	AUC
Xia, Zhang (40)	Cascaded multi-column RVFL neural network	105(56)	Cross-validation	83/22	No report
Xu, Zhao (41)	KNN, SVM, LR	122(92)	Train-Test Split	98/24	0.9260/0.9040
Zhang, Xie (44)	LR, RF, GBDT, LightGBM, XGBoost, CatBoos	719(255)	Cross-validation	575/144	No report
Maimaitituerxun, Chen (55)	DT	1001(274)	Random sampling	705/296	Decision tree:0.75/0.67;Logistic:0.76/0.69
Jiang, Liu (51)	LR	306(186)	Bootstrap	No	0.893/—(No report Independently verified AUC)
Ding, Yu (49)	LR	1256(412)	Random sampling	880/376	0.751/0.776
Yu, Jiang (45)	LR	530(179)	Cross-validation	414/116	0.914/0.859
Zhou, Dai (57)	LR	320(140)	Random sampling	224/96	0.816/0.858

RVFL, Random Vector Functional Link; KNN, k-NearestNeighbor; SVM, Support Vector Machine; LR, Logistic Regression; RF, Random Forest; GBDT, Gradient Boosting Decision Tree; LightGBM, a decision tree algorithm based on histograms; XGBoost, a boosting algorithm based on CART trees; CatBoost, an algorithm that utilizes symmetric decision trees (oblivious trees) as its base learner; DT, Decision Tree.

No statistical synthesis was performed across prevalence, risk factor, and prediction model studies, as their research objectives and outcome measures differed fundamentally.

## Discussion

This review synthesizes the prevalence, risk factors, and predictive models for CI in individuals with DM. The global pooled prevalence of CI was 40.80% (95% CI: 33.91–47.87). Several demographic, clinical, and metabolic factors, including age, low income, unmarried status, HbA1c, HCY, hypoglycemia, diabetic nephropathy, diabetic retinopathy, and stroke, were associated with an increased risk of CI. The discriminatory ability of the included predictive models was moderate to good. The pooled C-index values were 0.83 in training cohorts and 0.81 in validation cohorts. Nonetheless, given the limited number of studies, methodological heterogeneity, and lack of robust external validation, their clinical applicability should be interpreted with caution.

### Prevalence of CI in DM patients

This study revealed a relatively high pooled prevalence of CI among patients with DM. Previous research has similarly demonstrated cognitive decline in DM populations, particularly in the form of MCI, which is often regarded as an early stage of CI. For example, Zhao, Wang (13) reported an MCI prevalence of 44.1% in a cohort of 10,469 individuals with type 2 DM (T2DM), while a systematic review by You, Liu (5) found a prevalence of 45.0% among T2DM patients. These findings are consistent with our pooled estimate and further support the widespread nature of cognitive decline in individuals with DM.

Compared to previous meta-analyses, the present review provides several notable advancements. First, while studies such as You, Liu (5) and Zhao, Wang (13) focused solely on MCI, we included a broader range of CI stages, encompassing both mild and advanced cognitive dysfunction. Second, earlier reviews were largely based on data from China and other Asian countries, limiting their global applicability. In contrast, our study included data from six continents. However, the distribution of studies was uneven, with most studies from China. Third, while Zheng, Ma (63) primarily focused on identifying risk factors for CI, the present review extends the existing literature by integrating predictive modeling approaches—including both traditional regression and machine learning methods—alongside prevalence and risk analyses. By synthesizing evidence across these three domains, this study provides a more comprehensive and translational framework to inform early detection and intervention strategies in patients with DM.

Our findings also expand upon prior evidence from a large-scale meta-analysis by Xue, Xu (64), which demonstrated that DM significantly increases the risk of both CI and dementia, with pooled relative risks ranging from 1.25 to 1.91. In addition to prevalence-focused reviews, prior research has primarily examined relative risks rather than absolute prevalence estimates. Additionally, inconsistencies in diagnostic criteria and cognitive assessment tools across studies may have affected the accuracy of prevalence estimates. To address these limitations, our review incorporated broader regional data and conducted subgroup analyses based on geographic regions.

Significant regional variations were observed. Given the uneven distribution of studies across continents, these findings should be interpreted cautiously, particularly for regions represented by a single study. For instance, in Asia, the prevalence reached 70–80% in Saudi Arabia (30) and 54.51% in Thailand (38), while in Africa, Morocco reported a prevalence of 52.00% (34, 39)—all substantially higher than the overall pooled rate of 40.80%. These disparities may be influenced by variations in economic development, cultural and dietary practices, education levels, healthcare access, and diabetes management capabilities. Supporting this, Hill-Briggs, Adler (65) highlighted how social determinants—including food environments, socioeconomic status, and healthcare distribution—affect DM management and outcomes.

Furthermore, our study observed marked differences in the detection rate of CI across assessment tools. The prevalence of CI identified by the MoCA was 49.42%, notably higher than that detected by the MMSE (33.87%) and other instruments (29.40%). This difference likely reflects the superior sensitivity of the MoCA in detecting MCI. This finding is consistent with previous evidence, which demonstrates that MoCA more effectively distinguishes normal aging from early cognitive decline compared to MMSE (66). Therefore, variability in cognitive assessment instruments and diagnostic thresholds may represent an important source of methodological heterogeneity in this meta-analysis.

In addition, subgroup analyses stratified by demographic characteristics demonstrated that women, individuals aged > 60 years, those with ≤ 12 years of education, those without regular exercise, those unemployed, and those living in rural areas all exhibited an increased prevalence of CI. These findings align with previous evidence that lower educational attainment, disadvantaged socioeconomic conditions, and unhealthy lifestyles contribute to increased susceptibility to CI (67–69). Collectively, our findings suggest that the prevalence of CI in DM patients is influenced by an interplay of social, demographic, and methodological factors. Therefore, in clinical settings, it is crucial to select appropriate cognitive screening tools tailored to patient characteristics and to prioritize early identification and intervention in high-risk populations.

Notably, substantial heterogeneity was observed in the meta-analysis of the prevalence and subgroup analyses. This heterogeneity may be attributable to several factors, including differences in geographic regions, age distributions, educational backgrounds, and socioeconomic contexts, as well as variation in cognitive assessment tools and diagnostic thresholds. These findings align with previous evidence that lower educational attainment is associated with disadvantaged social status. Therefore, the pooled estimates should be interpreted as summary indicators reflecting an overall trend rather than precise global parameters. Future studies employing standardized diagnostic criteria and multicenter prospective designs are needed to reduce methodological heterogeneity and enhance comparability across populations.

### Risk factors associated with CI

In the analysis of risk factors for CI among individuals with DM, this study systematically examined four domains: demographic characteristics, lifestyle habits, diabetic complications, and metabolic or biochemical indicators. Among demographic factors, age consistently emerged as a significant risk factor. With advancing age, neurodegenerative changes, reduced vascular elasticity, and depletion of cognitive reserve collectively increase susceptibility to CI in DM patients. This finding aligns with those from multiple systematic reviews (13, 70). In addition, low educational level, unmarried or widowed status were also found to significantly elevate the risk of CI (71–73), suggesting that limited cognitive reserve, lack of social support, and psychosocial stress may be underlying mechanisms.

With regard to lifestyle factors, although existing studies have reported inconsistent findings on behavioral variables such as smoking and alcohol consumption, regular physical activity has been widely recognized as beneficial in delaying cognitive aging. Proposed mechanisms include improved insulin sensitivity, enhanced cerebral blood flow, and reduced systemic inflammation (74). In contrast, sedentary behavior and lack of exercise may exacerbate glucose dysregulation and increase the risk of neural damage.

Regarding clinical variables, diabetic nephropathy, history of hypoglycemia, and history of stroke were all significantly related to an elevated risk of CI. These findings suggest the potential role of both microvascular and macrovascular complications in impairing cerebrovascular structure and function (75, 76). Stroke was identified as one of the strongest risk factors, underscoring the long-term influence of cerebrovascular events on cognitive trajectories. This finding is consistent with previous systematic reviews and meta-analyses, which identified stroke as a significant, independent, and potentially modifiable risk factor for dementia (77).

Elevated HbA1c and HCY levels are significantly linked to a greater risk of CI, suggesting the roles of poor metabolic control in DM and chronic inflammation in neural damage (64, 78). Moreover, psychological status also warrants careful consideration, as depressive symptoms have been shown to co−occur with cognitive decline, potentially through activation of the hypothalamic–pituitary–adrenal axis and subsequent alterations in the expression of brain−derived neurotrophic factors (79).

Overall, the results of the present study highlight the multifactorial interactions underlying the risk of CI in DM patients, emphasizing the critical roles of age, social support, education level, metabolic indicators, and DM-related complications in the development of CI. Clinically, identifying modifiable and non-modifiable risk factors can facilitate stratified management, early screening, and individualized interventions. Nevertheless, most included studies were cross-sectional, limiting causal inference, and some variables demonstrated high heterogeneity. This underscores the need for more prospective studies with standardized cognitive assessment criteria to further validate and clarify the pathways through which these risk factors influence CI.

Furthermore, according to the GRADE assessment, the certainty of evidence for most identified risk factors ranged from low to very low. While associations such as educational attainment and marital status demonstrated relatively consistent estimates, the certainty of evidence for many other factors was downgraded due to substantial heterogeneity, imprecision, or limited numbers of studies. These findings suggest that although several risk factors appear to be associated with CI in patients with DM, the overall certainty in these associations remains limited. Therefore, the results should be interpreted with caution, and further well-designed prospective studies are warranted to strengthen the evidence.

### Predictive models

Among the included literature, 8 studies developed predictive models for CI in DM patients. These studies mainly employed traditional logistic regression methods and machine learning approaches. Overall, the models demonstrated good discriminative ability, with reported C-index or AUC values ranging from 0.67 to 0.93. Models based on logistic regression or nomograms generally performed better and were easier to interpret, whereas decision tree and imaging-based models tended to show lower stability. However, substantial heterogeneity in sample characteristics, variable selection, and modeling processes limited their stability and generalizability.

Although discrimination was frequently reported, only a few studies evaluated calibration performance (e.g., Hosmer–Lemeshow test or calibration curves), and even fewer conducted decision curve analyses (DCA) to assess clinical utility. None of the models underwent true multicenter external validation, indicating that their clinical applicability remains preliminary.

Systematic reviews specifically focusing on the early prediction of CI in diabetic populations remain limited. A recent review by Zhang et al. focused exclusively on MCI in T2DM patients, highlighting that most predictive models utilized common clinical and demographic variables, including age, education level, diabetes duration, HbA1c, and depression (14). However, reported AUC values varied significantly across studies, and many models were rated at high risk of bias due to limited sample size and lack of validation. The results of that review align with the current findings, suggesting that research in this field remains in its developmental stage.

From a clinical readiness perspective, several models, such as those proposed by Yu, Jiang (45) and Ding, Yu (49), demonstrated relatively high applicability because they used easily accessible predictors (e.g., age, HbA1c, physical activity, social support) and performed internal or temporal validation. However, models requiring advanced biomarkers (e.g., Galectin-3) or neuroimaging features may face challenges in real-world implementation due to high costs and limited accessibility. Existing research remains limited by the small number of available prediction model studies, a low diversity of predictors, and the lack of robust external validation. Therefore, future research should rely on large, multicenter prospective cohorts, incorporating additional lifestyle factors, microvascular complications, inflammatory markers, and neuroimaging features, while applying rigorous internal cross-validation and external validation to enhance model generalizability and clinical applicability.

Overall, the discrepancies observed across prevalence estimates, risk associations, and predictive model performance are likely influenced by substantial methodological heterogeneity among the included studies. Differences in cognitive assessment instruments, diagnostic thresholds, and case definitions may have directly influenced both prevalence and risk estimates. Moreover, the predominance of studies from specific geographic regions, particularly Asia, may have affected pooled estimates, given variations in socioeconomic conditions, healthcare systems, DM management practices, and educational attainment across populations. Heterogeneity in study design, sample size, covariate adjustment strategies, and model validation procedures further affects the direct comparability between studies. Therefore, while the present review provides a synthesis of existing evidence, the findings should be interpreted with caution, and the generalizability of the results across diverse settings is limited.

### Early intervention implications

In addition to early prediction, interventions are equally critical for slowing cognitive decline. Existing evidence indicates that various non-pharmacological interventions can effectively delay cognitive decline among older individuals. A systematic review and meta-analysis by Chen et al. (80) demonstrated that Tai Chi notably improved executive function, episodic memory, visuospatial ability, and overall cognition in older adults with MCI. Similarly, Li et al. (81), in a meta-analysis involving over 4,400 older adults, found that structured cognitive training markedly improved cognitive performance across multiple domains, provided that participant engagement, adherence, and compliance were high. These findings suggest that in high-risk DM populations, developing and applying scientifically robust risk prediction models can not only facilitate early screening but also provide evidence-based guidance for individualized interventions (such as Tai Chi or cognitive training), thereby delaying the onset and progression of CI.

### Strengths and limitations of the study

This systematic review comprehensively assessed the prevalence and risk factors of CI in DM patients, with a particular focus on summarizing the performance of early predictive models. A major strength of this study lies in that it comprehensively integrates epidemiological burden, risk determinants, and the performance metrics of prediction models. By synthesizing these traditionally separate domains, the present review offers a more coherent foundation for early screening and intervention strategies of CI. Our findings may offer important insights for the diagnosis and prevention of CI in this population. However, certain limitations should be acknowledged. First, substantial heterogeneity was observed across studies, likely reflecting both genuine differences in population characteristics and methodological variations, including geographic region, age distribution, cognitive assessment tools, and covariate adjustment. Therefore, the pooled estimates should be interpreted as approximate summary estimates rather than precise global parameters. Second, although studies were conducted across multiple continents, most studies originated from China and India, and other regions were underrepresented, which may limit the global generalizability of the findings. Third, given the predominance of cross-sectional studies, it is impossible to infer causal relationships. Finally, non-English studies and gray literature were excluded, which may have introduced language and publication bias.

## Conclusions

The global prevalence of CI among DM patients is high and varies substantially across regions and assessment tools. Advanced age, low income, depression, diabetic retinopathy, diabetic nephropathy, hypoglycemia, homocysteine, and stroke were identified as significant risk factors for CI. Importantly, several of these factors are potentially modifiable. Accordingly, tight glycemic control, the prevention of vascular complications, and psychosocial support may be crucial for reducing the risk of CI. Although the discriminatory performance of predictive models is moderate to promising, these tools remain in an exploratory stage, given the limited number of studies, methodological heterogeneity, and the lack of robust external validation. These findings indicate that it is important to raise the awareness of CI in individuals with DM, and these predictive models should be used cautiously in clinical practice. Strengthening public health strategies and promoting multidisciplinary approaches may help inform future prevention and management strategies for individuals with DM.
