# Supplementary material for: Prevalence, risk factors, and early prediction of cognitive impairment in patients with diabetes mellitus: a systematic review and meta-analysis
Source: Front Endocrinol (Lausanne). 2026 Mar 25;17:1757367. doi: 10.3389/fendo.2026.1757367 (PMC13056624; doi:10.3389/fendo.2026.1757367)
Supplement: Supplementary file 1 [file DataSheet1.pdf]

SUPPLEMENTARY FILE CONTENT

TITLE	PAGE
**Supplementary Table S1.** Search strategy	1-3
**Supplementary Table S2.** Inclusion of 41 studies characteristics	4-11
**Supplementary Table S3.** Quality assessment results of cross-sectional studies using AHRQ	12-14
**Supplementary Table S4.** Quality assessment results of case-control and cohort studies using NOS	14
**Supplementary Figure S1** Forest plot of assessment and diagnostic tools	15
**Supplementary Figure S2** Forest plots by continents	16
**Supplementary Figure S3** Funnel Plot Adjusted Using Trim-and-Fill Method	17
**Supplementary Figure S4-30** Synthesized effect size of risk factors for CI in DM	18

Supplementary Table S1 Search strategies

Database	Search strategies	Search results
WOS	#1<br>TS=(Diabetes Mellitus or Type 2 Diabetes or T2DM or Type 1 Diabetes or Diabetes or diabetic or diabets)<br>#2<br>TS=(Cognitive Dysfunction or Cognitive Dysfunctions or Cognitive Impairments or Cognitive Impairment or Cognitive Disorder or Cognitive Disorders or Mild Cognitive Impairment or Mild Cognitive Impairments or Cognitive Decline or Cognitive Declines or Mental Deterioration or Mental Deteriorations)<br>#3<br>TS=(machine learning or artificial intelligence or Transfer Learning or Deep learning or Ensemble Learning or prediction model or risk model or risk score or random forest or neural network or neural networks or CNN or K-Nearest Neighbor or Support vector machine or SVM or Gradient Boosting Machine or Nomogram or XGBoost or Adaboost or LightGBM or CatBoost or Gradient Boosting or Decision tree or Regression Trees or ResNet or AlexNet or VGGNet or GoogLeNet or Naive Bayesian or Multilayer perceptron or Bayesian network or Radiomics or Radiomic or radiomics-based or radiomic signature or Texture or Risk factors or Predictors or biomarkers)<br>#1 AND #2 AND #3	7747
Pubmed	#1<br>("Diabetes Mellitus"[Mesh]) OR ((((((Type 2 Diabetes[Title/Abstract]) OR (T2DM[Title/Abstract])) OR (Type 1 Diabetes[Title/Abstract])) OR (Diabetes[Title/Abstract])) OR (diabetic[Title/Abstract])) OR (diabets[Title/Abstract]))<br>#2<br>("Cognitive Dysfunction"[Mesh]) OR (((((((((((Cognitive Dysfunctions[Title/Abstract]) OR (Cognitive Impairments[Title/Abstract])) OR (Cognitive Impairment[Title/Abstract])) OR (Cognitive Disorder[Title/Abstract])) OR (Cognitive Disorders[Title/Abstract])) OR (Mild Cognitive Impairment[Title/Abstract])) OR (Mild Cognitive Impairments[Title/Abstract])) OR (Cognitive Decline[Title/Abstract])) OR (Cognitive Declines[Title/Abstract])) OR (Mental Deterioration[Title/Abstract])) OR (Mental Deteriorations[Title/Abstract]))<br>#3	2267

	("Machine Learning"[Mesh]) OR ((((((((((((((((((((((((((((((((((((((artificial intelligence[Title/Abstract]) OR (Transfer Learning[Title/Abstract])) OR (Deep learning[Title/Abstract])) OR (Ensemble Learning[Title/Abstract])) OR (prediction model[Title/Abstract])) OR (risk model[Title/Abstract])) OR (risk score[Title/Abstract])) OR (random forest[Title/Abstract])) OR (neural network[Title/Abstract])) OR (neural networks[Title/Abstract])) OR (CNN[Title/Abstract])) OR (K-Nearest Neighbor[Title/Abstract])) OR (Support vector machine[Title/Abstract])) OR (SVM[Title/Abstract])) OR (Gradient Boosting Machine[Title/Abstract])) OR (Nomogram[Title/Abstract])) OR (XGBoost[Title/Abstract])) OR (Adaboost[Title/Abstract])) OR (LightGBM[Title/Abstract])) OR (CatBoost[Title/Abstract])) OR (Gradient Boosting[Title/Abstract])) OR (Decision tree[Title/Abstract])) OR (Regression Trees[Title/Abstract])) OR (ResNet[Title/Abstract])) OR (AlexNet[Title/Abstract])) OR (VGGNet[Title/Abstract])) OR (GoogLeNet[Title/Abstract])) OR (Naive Bayesian[Title/Abstract])) OR (Multilayer perceptron[Title/Abstract])) OR (Bayesian network[Title/Abstract])) OR (Radiomics[Title/Abstract])) OR (Radiomic[Title/Abstract])) OR (radiomics-based[Title/Abstract])) OR (radiomic signature[Title/Abstract])) OR (Texture[Title/Abstract])) OR (Risk factors[Title/Abstract])) OR (Predictors[Title/Abstract])) OR (biomarkers[Title/Abstract]))<br>#1 AND #2 AND #3	
Cochrane	#1 MeSH descriptor: [Diabetes Mellitus] explode all trees<br>#2 (Type 2 Diabetes or T2DM or Type 1 Diabetes or Diabetes or diabetic or diabets): ti,ab,kw<br>#3 #1 or #2<br>#4 MeSH descriptor: [Cognitive Dysfunction] explode all trees<br>#5 (Cognitive Dysfunctions or Cognitive Impairments or Cognitive Impairment or Cognitive Disorder or Cognitive or Disorders or Mild Cognitive Impairment or Mild Cognitive Impairments or Cognitive Decline or Cognitive Declines or Mental Deterioration or Mental Deteriorations) : ti,ab,kw<br>#6 #4 or #5<br>#7 MeSH descriptor: [machine learning] explode all trees<br>#8 (artificial intelligence or Transfer Learning or Deep learning or Ensemble Learning or prediction model or risk model or risk score or random forest or neural network or neural networks or CNN or K-Nearest Neighbor or Support vector or machine or SVM or Gradient Boosting Machine or Nomogram or XGBoost or Adaboost or LightGBM or CatBoost or Gradient Boosting or Decision tree or	185

	Regression Trees or ResNet or AlexNet or VGGNet or GoogLeNet or Naiveor Bayesian or Multilayer perceptron or Bayesian network or Radiomics or Radiomic or radiomics-based or radiomic or signature or Texture or Risk factors or Predictors or biomarkers): ti,ab,kw<br>#9 #7 or #8<br>#3 AND #6 AND #9	
Embase	#1<br>'diabetes mellitus'/exp OR 'diabetes mellitus': ab,ti OR 'type 2 diabetes':ab,ti OR t2dm:ab,ti OR 'type 1 diabetes':ab,ti OR diabetes:ab,ti OR diabetic:ab,ti OR diabetics:ab,ti<br>#2<br>'cognitive defect'/exp OR 'cognitive defect':ab,ti OR 'cognitive defects':ab,ti OR 'cognitive disability':ab,ti OR 'cognitive dysfunction':ab,ti OR overinclusion:ab,ti OR 'response interference':ab,ti<br>#3<br>'machine learning'/exp OR 'machine learning':ab,ti OR 'artificial intelligence':ab,ti OR 'transfer of learning':ab,ti OR 'deep learning':ab,ti OR 'ensemble learning':ab,ti OR 'prediction model':ab,ti OR 'risk model':ab,ti OR 'risk score':ab,ti OR 'random forest':ab,ti OR 'neural network':ab,ti OR 'neural networks':ab,ti OR cnn:ab,ti OR 'k-nearest neighbor':ab,ti OR 'support vector machine':ab,ti OR svm:ab,ti OR 'gradient boosting machine':ab,ti OR nomogram:ab,ti OR xgboost:ab,ti OR 'adaboost':ab,ti OR lightgbm:ab,ti OR catboost:ab,ti OR 'gradient boosting':ab,ti OR 'decision tree':ab,ti OR 'regression trees':ab,ti OR 'residual neural network':ab,ti OR alexnet:ab,ti OR vggnet:ab,ti OR googlenet:ab,ti OR 'naive bayesian':ab,ti OR 'multilayer perceptron':ab,ti OR 'bayesian network':ab,ti OR radiomics:ab,ti OR 'radiomics':ab,ti OR 'radiomics based':ab,ti OR 'radiomic signature':ab,ti OR texture:ab,ti OR 'risk factors':ab,ti OR predictors:ab,ti OR biomarkers:ab,ti<br>#1 AND #2 AND #3	9383

Supplementary Table S2 Inclusion of 41 studies characteristics

No	Study: Author （year）	Country	Study of type	Diagnosis tool	Mean age	Gender, % Female	Outcome	DM cases	Risk factors	Type of prediction	Quality score
1	Bruce et al. (2008)	Australia	A cohort Study	MMSE	76.0±4.6	156 (51.66)	CI 60<br>DM 28	302	①②⑦⑧⑨	None	9
2	Chen et al. (2012)	China	A cross-sectional study	MoCA	55.34 ± 8.35	76 (48.41)	MCI 93	157	NA	None	5
3	Lavielle et al. (2015)	Mexico	A cross-sectional study	others	51.00 ± 11.00	1083 (63.26)	CI 38	1712	NA	None	8
4	Moulton et al. (2016)	UK	A cross-sectional study	others	56.10±11.00	740 (44.05)	CI 176	980	①②	None	7
5	Gao et al. (2016)	China	A cross-sectional study	others	72.40± 3.50	624 (56.27)	MCI 690<br>DM 132	1109	⑤⑦⑧⑰⑱⑳㉒	None	8
6	Murata et al. (2017)	Japan	A cross-	MMSE	73.40± 6.10	88 (44.90)	MCI 73<br>DM 15	196	NA	None	5

			section al study								
7	Blanquisco et al. (2017)	Philippin es	A cross-section al study	MoCA	67.00 ±4.80	93 (69.92)	MCI 60	133	①②⑤⑦ ⑭ ⑮⑱⑳㉕	None	7
8	Abba et al. (2018)	Cameroo n	A cross-section al study	MMSE	56.00± 9.50	121 (54.26)	CI 33	223	NA	None	7
9	Li et al. (2019)	China	A cross-section al Study	others	74.23 ± 6.93	277 (57.11)	MCI 56	485	③⑩	None	7
10	Xiu et al. (2019)	China	A cross-section al Study	MMSE	71.21 ± 7.41	1653 (62.95)	CI 260	262 6	⑩⑯㉓㉕	None	6
11	Naguib et al. (2020)	Kingdom of Saudi Arabia	A cross-section al study	MoCA	56.01 ± 9.97	185 (68.8)	CI 216	269	①②④⑦ ㉖ ㉗	None	6

12	Sun et al. (2020)	China	A cross-sectional study	MoCA	64:43 ± 15:29	38 (31.67)	CI 80	120	②⑤⑦⑨⑰⑱㉑㉒	None	5
13	Xia et al. (2020)	China	A cross-sectional study	MoCA	56.80±6.90	105 (17.77)	MCI 174<br>DM 47	591	②⑥⑧⑫⑬	None	7
14	Fu et al. (2021)	China	A case–control study	others	65.87±5.37	62 (43.97)	CI 65	141	②③⑦⑱⑲	None	8
15	Althubaity et al. (2021)	Kingdom of Saudi Arabia	A cross-sectional study	MoCA	58.60±9.60	NA	MCI 119	179	②④⑭	None	7
16	Suain Bon et al. (2021)	Malaysia	A cross-sectional study	MoCA	68.40 (6.00)	56 (49.56)	CI 53	113	①② ⑭㉔㉖	None	7
17	Subramanian et al. (2021)	India	A cross-sectional study	MMSE	63.90 ± 7.10	150 (62.50)	CI 72	240	①②⑥⑮㉕㉖	None	7
18	Xu et al. (2021)	China	A cross-	MoCA	61.15 ± 8.15	85 (35.42)	CI 126	240	②⑩⑮㉓	None	6

			section al study								
19	Bozanic et al. (2022)	Chile	A cross-section al study	others	70.80 ± 4.50	40 (64.52)	CI 19	62	③⑦	None	7
20	Panyawattanakit et al. (2022)	Thailand	A cross-section al study	others	IQR: 71 (65–75)	153 (62.70)	CI 133	244	①②⑪㉗	None	6
21	Rhmari Tlemçani et al. (2022)	Morocco	A cohort study	MMSE	IQR: 59-70	35 (35.00)	CI 53	100	②	None	7
22	Xia et al. (2022)	China	A cross-section al study	MoCA	64.69±7.22	53 (50.48)	MCI 56	105	⑫	RVFL	5
23	Xu et al. (2022)	China	A cross-section al study	MoCA	DM：65.08 ± 7.38 MCI：62.67 ± 6.90	83 (68.03)	DM 40 MCI 52	122	NA	Radiomics + LR	7
24	Anand et al. (2023)	India	A cross-	MoCA	NA	67 (39.41)	CI 17	170	NA	None	6

			section al study								
25	Ma et al. (2023)	China	A case–control study	MMSE	IQR: 67 (61, 74)	57 (11.31)	CI 280	504	⑲㉔	None	8
26	Zhang et al. (2023)	China	A cross-section al study	MoCA	64.32±8.3 2	340 (47.29)	CI 255	719	NA	Six machine learning models: LR, RF, GBDT, LightGBM, XGBoost, CatBoost	7
27	Yu et al. (2024)	China	A cross-section al study	MoCA	NA	297 (56.04)	MCI 179	530	②④⑤⑩㉔	LR+Nomogra m	7
28	Luo et al. (2024)	China	A cross-section al study	MoCA	60.62	47 (45.63)	MCI 50	103	③㉑	None	7
29	Ashebir et al. (2024)	Ethiopia	A cross-section al study	MMSE	NA	169 (40.14)	CI 184	421	⑥㉗	None	8

30	Carandang et al. (2024)	Philippine	A cross-sectional study	others	56.30 ± 9.70	103 (60.23)	CI 33	171	①②⑤⑦⑨⑭⑱	None	7
31	Ding et al. (2024)	China	A cross-sectional study	MoCA	57.5 0± 11.20	427 (34.00)	MCI 412	1256	②③⑮⑱	LR	7
32	Getasew Hiruy et al. (2024)	Ethiopia	A cross-sectional study	MMSE	56.00 ± 11.15	195 (48.03)	CI 112	406	①②⑥⑦ ⑪㉓㉕	None	7
33	Jiang et al. (2024)	China	A cross-sectional study	MoCA	66.19 ± 1.92	131 (42.81)	CI 186	306	①⑥⑦⑩⑱	LR+Nomogram	6
34	Li et al. (2024)	China	A cross-sectional study	MoCA	IQR :59.5 (56.0 to 62.0)	239 (45.61)	CI 204	524	②③⑧⑮	None	7
35	M. Liu et al. (2024)	China	A cross-sectional study	MMSE	61.73±9.08	129 (41.88)	CI 125	308	①	None	7
36	X. Liu et al. (2024)	China	A cross-	MoCA	69.72±5.9	576 (64.14)	CI 451	898	①②⑥⑦⑩	None	9

			section al study								
37	Maimaitituerxun et al. (2024)	China	A case–control study	MMSE	NA	388 (38.76)	MCI 274	1001	①②⑥⑤⑦ ⑫ ⑬ ⑭ ⑯ ㉓㉕㉖㉗	A CHAID decision tree	8
38	H. Liu et al. (2024)	China	A cross-section al study	MMSE	69.80 ± 9.10	277 (58.44%)	CI 187	475	②⑯㉗	None	9
39	Zhou et al. (2025)	China	A cross-section al study	MoCA	NA	109 (34.06)	MCI 140	320	①②③⑦	LR+Nomogram	9
40	Raghuveer et al. (2025)	India	A cross-section al study	MoCA	52.94 ± 12.24	35 (32.41)	CI 63	108	①②⑭㉖	None	6
41	Khan et al. (2025)	India	A cross-section al study	MMSE	66.68±5.56	38(38.38%)	CI 37	99	②⑱㉓㉕	None	6

①sex, ②age ③education level ④exercise, ⑤smoking, ⑥alcohol use ⑦DM duration, ⑧ overweigh ⑨body mass index [BMI],

⑩depression ⑪ Comorbidities ⑫ diabetic nephropathy, ⑬ diabetic retinopathy, ⑭ hypertension, ⑮ hypoglycemia, ⑯ stroke ⑰ fasting plasma glucose [FPG] ⑱ hemoglobin A1c [HbA1c] ⑲ homocysteine [HCY] ⑳ high-density lipoprotein [HDL] ㉑ Homeostatic Model Assessment of Insulin Resistance [HOMA-IR] ㉒ TC（Total Cholesterol）㉓ Living area ㉔ Living alone ㉕ Marital status ㉖ Employment ㉗ Income

Note: MMSE Mini-mental state exam, MoCA Montreal Cognitive Assessment, DM Diabetes Mellitus, CI Cognitive impairment, MCI Mild cognitive impairment, LR Logistic regression, RVFL c , RF Random Forest, GBDT Gradient Boosting Decision Tree, LightGBM A decision tree algorithm based on histograms,, XGBoost A boosting algorithm based on CART trees, CatBoost An algorithm that utilizes symmetric decision trees (oblivious trees) as its base learner, CHAID Chi-square automatic interaction detection , CNN A convolutional neural network

Supplementary Table S3 Quality assessment results of cross-sectional studies using AHRQ

**Cross-sectional study/No.**	**Study: Author (year)**	**v1**	**v2**	**v3**	**v4**	**v5**	**v6**	**v7**	**v8**	**v9**	**v10**	**v11**	**Total Score**	**quality level**
1	Chen et al. (2012)	Y	Y	Y	UN	N	UN	Y	Y	N	N	NA	5	Moderate
2	Lavielle et al. (2015)	Y	Y	Y	Y	N	Y	Y	Y	N	Y	NA	8	High
3	Moulton et al. (2016)	Y	Y	Y	N	N	N	Y	Y	Y	Y	NA	7	Moderate
4	Gao et al. (2016)	Y	Y	Y	Y	N	Y	Y	Y	N	Y	NA	8	High
5	Murata et al. (2017)	Y	Y	Y	UN	N	Y	Y	Y	UN	UN	NA	5	Moderate
6	Blanquisco et al. (2017)	Y	Y	Y	Y	N	N	Y	Y	N	Y	NA	7	Moderate
7	Abba et al. (2018)	Y	Y	Y	Y	N	N	Y	Y	N	Y	NA	7	Moderate
8	Li et al. (2019)	Y	Y	N	Y	Y	N	Y	Y	N	Y	NA	7	Moderate
9	Xiu et al. (2019)	Y	Y	Y	Y	N	N	Y	Y	N	N	NA	6	Moderate
10	Naguib et al. (2020)	Y	Y	Y	N	N	N	Y	Y	N	Y	NA	6	Moderate
11	Sun et al. (2020)	Y	Y	Y	N	N	N	Y	Y	N	N	NA	5	Moderate
12	Xia et al. (2020)	Y	Y	Y	N	Y	N	Y	Y	N	Y	NA	7	Moderate
13	Althubaity et al. (2021)	Y	Y	Y	Y	N	N	Y	Y	N	Y	NA	7	Moderate
14	Suain Bon et al. (2021)	Y	Y	Y	Y	N	N	Y	Y	N	Y	NA	7	Moderate
15	Subramanian et al. (2021)	Y	Y	Y	Y	N	N	Y	Y	N	Y	NA	7	Moderate
16	Xu et al. (2021)	Y	Y	Y	N	N	N	Y	Y	N	Y	NA	6	Moderate
17	Bozanic et al. (2022)	Y	Y	Y	Y	N	N	Y	Y	N	Y	NA	7	Moderate
18	Panyawattanakit et al. (2022)	Y	Y	Y	N	N	N	Y	Y	N	Y	NA	6	Moderate
19	Xia et al. (2022)	Y	Y	N	N	N	N	Y	Y	N	Y	NA	5	Moderate

20	Xu et al. (2022)	Y	Y	Y	N	Y	Y	Y	N	N	Y	NA	7	Moderate
21	Zhang et al. (2023)	Y	Y	Y	N	N	N	Y	Y	Y	Y	NA	7	Moderate
22	Anand et al. (2023)	Y	Y	Y	N	N	N	Y	Y	N	Y	NA	6	Moderate
23	Yu et al. (2024)	Y	Y	Y	Y	N	N	Y	Y	N	Y	NA	7	Moderate
24	Luo et al. (2024)	Y	Y	Y	N	N	N	Y	Y	Y	Y	NA	7	Moderate
25	Ashebir et al. (2024)	Y	Y	Y	Y	N	Y	Y	Y	N	Y	NA	8	High
26	Carandang et al. (2024)	Y	Y	Y	N	N	N	Y	Y	Y	Y	NA	7	Moderate
27	Ding et al. (2024)	Y	Y	Y	Y	N	N	Y	Y	Y	Y	NA	8	High
28	Getasew Hiruy et al. (2024)	Y	Y	Y	Y	N	N	Y	Y	N	Y	NA	7	Moderate
29	Jiang et al. (2024)	Y	Y	Y	N	N	N	Y	Y	N	Y	NA	6	Moderate
30	Li et al. (2024)	Y	Y	Y	Y	N	N	Y	Y	N	Y	NA	7	Moderate
31	M. Liu et al. (2024)	Y	Y	Y	N	N	N	Y	Y	N	Y	NA	7	Moderate
32	X. Liu et al. (2024)	Y	Y	N	Y	Y	Y	Y	Y	Y	Y	NA	9	High
33	Zhou et al. (2025)	Y	Y	Y	Y	N	Y	Y	Y	Y	Y	NA	9	High
34	H. Liu et al. (2024)	Y	Y	Y	Y	N	Y	Y	Y	Y	Y	NA	9	High
35	Raghuveer et al. (2025)	Y	Y	Y	N	N	N	Y	Y	N	Y	NA	6	Moderate
36	Khan et al. (2025)	Y	Y	Y	N	N	N	Y	Y	N	Y	NA	6	Moderate

Note: The AHRQ criteria for evaluating the quality of cross-sectional studies include a total of 11 items, each evaluated using “Yes,” “No,” or “Unclear”: V1(Define the source of information (survey, record review),V2(List inclusion and exclusion criteria for exposed and unexposed subjects (cases and controls) or refer to previous publications),V3(Indicate time period used for identifying patients.), V4(Indicate whether or not subjects were consecutive if not population-based), V5(Indicate if evaluators of subjective components of study were masked to other aspects of the participants), V6(Describe any assessments undertaken for quality assurance purposes (e.g.,test/retest of primary outcome measurements), V7(Explain any patient exclusions from analysis), V8(Describe how confounding was assessed and/or controlled),V9(If applicable, explain how missing data were handled in the analysis), V10(Summarize patient response rates and completeness of data collection), V11(Clarify what follow-

up, if any, was expected and the percentage of patients for which incomplete data or follow-up was obtained).

Supplementary Table S4 Quality assessment results of case-control and cohort studies using NOS

**A cohort Study/No.**	**Study: Author (year)**	**A1**	**A2**	**3**	**4**	**5**	**6**	**7**	**8**	**Total Score**	**Quality level**
1	Bruce et al. (2008)	1	1	1	1	1	1	1	1	8	high
2	Rhmari Tlemçani et al. (2022)	1	1	1	1	1	1	0	0	7	high
**A case–control study/No.**		**B1**	**B2**	**B3**	**B4**	**B5**	**B6**	**B7**	**B8**	**Total Score**	**Quality level**
1	Fu et al. (2021)	1	1	1	1	1	1	1	0	8	high
2	Ma et al. (2023)	1	1	1	1	1	1	1	0	8	high
3	Maimaitituerxun et al. (2024)	1	1	1	1	1	1	1	0	8	high

Study or Subgroup	Events	Total	Weight (common)	Weight (random)	IV, Fixed + Random, 95% CI
**diagnosis = MMSE**					
Bruce, D. G. (2008)	88	302	1.8%	2.6%	0.291 [0.241; 0.346]
Yuko Murata (2017)	88	281	1.7%	2.6%	0.313 [0.259; 0.371]
Zainab I. Abba (2018)	33	223	1.3%	2.6%	0.148 [0.106; 0.203]
Manimozhi Subramanian (2021)	72	240	1.4%	2.6%	0.300 [0.244; 0.363]
Fatima Zahra Rhmari Tlemçani (2022)	53	108	0.6%	2.6%	0.491 [0.394; 0.588]
Nimona Ashebir (2024)	184	421	2.5%	2.7%	0.437 [0.389; 0.486]
Endalk Getasew Hiruy (2024)	112	406	2.4%	2.7%	0.276 [0.232; 0.321]
Minli Liu (2024)	125	308	1.8%	2.7%	0.406 [0.350; 0.463]
Hongying Liu (2024)	187	475	2.8%	2.7%	0.394 [0.349; 0.439]
Fatima Khan (2025)	37	99	0.6%	2.6%	0.374 [0.281; 0.477]
**Total (common effect, 95% CI)**		**2863**	**16.9%**	.	**0.339 [0.321; 0.356]**
**Total (random effect, 95% CI)**		.		**26.3%**	**0.338 [0.277; 0.401]**
Heterogeneity: $Tau^2 = 0.0101$; $Chi^2 = 98.31$, df = 9 (P < 0.0001); $I^2 = 90.8\%$					
**diagnosis = MoCA**					
Rui-Hua Chen (2012)	93	157	0.9%	2.6%	0.592 [0.511; 0.670]
Louren Blanquisco (2017)	60	133	0.8%	2.6%	0.451 [0.365; 0.540]
Rania Naguib (2020)	216	269	1.6%	2.6%	0.803 [0.750; 0.849]
Lin Sun (2020)	80	120	0.7%	2.6%	0.667 [0.575; 0.749]
San-Shan Xia (2020)	214	297	1.8%	2.6%	0.721 [0.667; 0.772]
Shaimaa K. Althubaity (2021)	119	179	1.1%	2.6%	0.665 [0.590; 0.733]
Rosnadia Suainbon (2021)	53	113	0.7%	2.6%	0.469 [0.375; 0.565]
Weiran Xu (2021)	126	240	1.4%	2.6%	0.525 [0.459; 0.591]
Shuiwei Xia (2022)	56	105	0.6%	2.6%	0.533 [0.434; 0.631]
Zhigao Xu (2022)	92	122	0.7%	2.6%	0.754 [0.668; 0.828]
Xin Zhang (2023)	255	719	4.2%	2.7%	0.355 [0.318; 0.390]
R.Vijay Anand (2023)	17	170	1.0%	2.6%	0.100 [0.058; 0.154]
Qian Yu (2024)	179	530	3.1%	2.7%	0.338 [0.298; 0.380]
Tao Luo (2024)	50	103	0.6%	2.6%	0.485 [0.386; 0.586]
Qun Ding (2024)	412	1256	7.4%	2.7%	0.328 [0.302; 0.355]
Yuanyuan Jiang (2024)	186	306	1.8%	2.7%	0.608 [0.551; 0.664]
Yao-Shuang Li (2024)	204	524	3.1%	2.7%	0.389 [0.347; 0.433]
Xueyan Liu (2024)	451	898	5.3%	2.7%	0.502 [0.469; 0.536]
Pracheth Raghuveer (2025)	63	108	0.6%	2.6%	0.583 [0.485; 0.676]
Xueling Zhou (2025)	140	320	1.9%	2.7%	0.438 [0.382; 0.494]
**Total (common effect, 95% CI)**		**6669**	**39.4%**	.	**0.458 [0.446; 0.470]**
**Total (random effect, 95% CI)**		.		**52.5%**	**0.513 [0.434; 0.592]**
Heterogeneity: $Tau^2 = 0.0314$; $Chi^2 = 672.63$, df = 19 (P < 0.0001); $I^2 = 97.2\%$					
**diagnosis = Others**					
Pilar Lavielle (2015)	38	1712	10.1%	2.7%	0.022 [0.014; 0.029]
Yuxia Gao (2016)	822	1109	6.5%	2.7%	0.741 [0.715; 0.767]
Calum D. Moulton (2016)	176	980	5.8%	2.7%	0.180 [0.155; 0.204]
Wei Li (2019)	56	485	2.9%	2.7%	0.115 [0.089; 0.148]
Shuangling Xiu (2019)	260	2626	15.5%	2.7%	0.099 [0.087; 0.110]
Agnieszka Bozanic Leal (2022)	19	62	0.4%	2.5%	0.306 [0.198; 0.437]
Chuthiphun Panyawattanakit (2022)	133	244	1.4%	2.6%	0.545 [0.480; 0.609]
Russell Anne Marie L (2024)	33	171	1.0%	2.6%	0.193 [0.135; 0.259]
**Total (common effect, 95% CI)**		**7389**	**43.6%**	.	**0.171 [0.163; 0.180]**
**Total (random effect, 95% CI)**		.		**21.2%**	**0.247 [0.097; 0.437]**
Heterogeneity: $Tau^2 = 0.0838$; $Chi^2 = 2472.81$, df = 7 (P = 0); $I^2 = 99.7\%$					
**Total (common effect, 95% CI)**		**16921**	**100.0%**	.	**0.305 [0.298; 0.312]**
**Total (random effect, 95% CI)**		.		**100.0%**	**0.408 [0.339; 0.479]**
Heterogeneity: $Tau^2 = 0.0485$; $Chi^2 = 4661.45$, df = 37 (P = 0); $I^2 = 99.2\%$					
Test for subgroup differences (common effect): $Chi^2 = 1417.70$, df = 2 (P < 0.0001)					
Test for subgroup differences (random effects): $Chi^2 = 14.07$, df = 2 (P = 0.0009)					

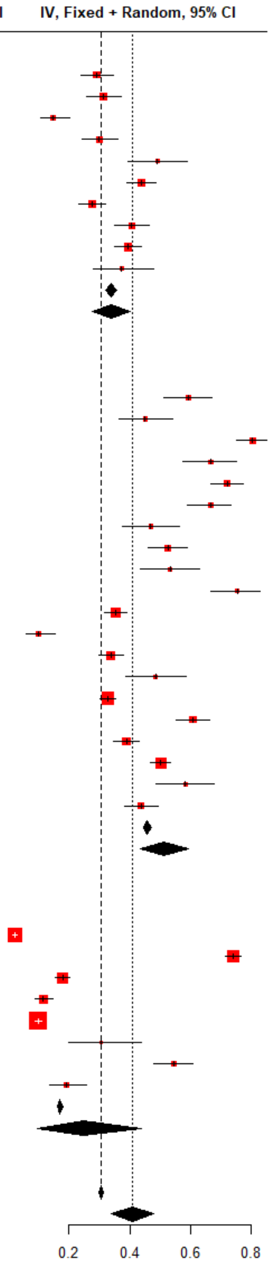

Supplementary Figure S1 Forest plot of assessment and diagnostic tools

Study or Subgroup	Events	Total	Weight (common)	Weight (random)	IV, Fixed + Random, 95% CI
**continent = Oceania**					
Bruce, D. G. (2008)	88	302	1.8%	2.6%	0.291 [0.241; 0.346]
**continent = Asia**					
Rui-Hua Chen (2012)	93	157	0.9%	2.6%	0.592 [0.511; 0.670]
Yuxia Gao (2016)	822	1109	6.5%	2.7%	0.741 [0.714; 0.767]
Yuko Murata (2017)	88	281	1.7%	2.6%	0.313 [0.260; 0.371]
Louren Blanquisco (2017)	60	133	0.8%	2.6%	0.451 [0.366; 0.539]
Wei Li (2019)	56	485	2.9%	2.7%	0.115 [0.088; 0.147]
Shuangling Xiu (2019)	260	2626	15.5%	2.7%	0.099 [0.085; 0.108]
Rania Naguib (2020)	216	269	1.6%	2.6%	0.803 [0.751; 0.849]
Lin Sun (2020)	80	120	0.7%	2.6%	0.667 [0.574; 0.749]
San-Shan Xia (2020)	214	297	1.8%	2.6%	0.721 [0.665; 0.770]
Shaimaa K. Althubaity (2021)	119	179	1.1%	2.6%	0.665 [0.590; 0.733]
Rosnadia Suainbon (2021)	53	113	0.7%	2.6%	0.469 [0.375; 0.565]
Manimozhi Subramanian (2021)	72	240	1.4%	2.6%	0.300 [0.243; 0.362]
Weiran Xu (2021)	126	240	1.4%	2.6%	0.525 [0.460; 0.590]
Chuthiphun Panyawattanakit (2022)	133	244	1.4%	2.6%	0.545 [0.480; 0.609]
Shuiwei Xia (2022)	56	105	0.6%	2.6%	0.533 [0.434; 0.631]
Zhigao Xu (2022)	92	122	0.7%	2.6%	0.754 [0.667; 0.826]
Xin Zhang (2023)	255	719	4.2%	2.7%	0.355 [0.317; 0.389]
R.Vijay Anand (2023)	17	170	1.0%	2.6%	0.100 [0.060; 0.156]
Qian Yu (2024)	179	530	3.1%	2.7%	0.338 [0.296; 0.379]
Tao Luo (2024)	50	103	0.6%	2.6%	0.485 [0.386; 0.586]
Russell Anne Marie L (2024)	33	171	1.0%	2.6%	0.193 [0.136; 0.260]
Qun Ding (2024)	412	1256	7.4%	2.7%	0.328 [0.302; 0.355]
Yuanyuan Jiang (2024)	186	306	1.8%	2.7%	0.608 [0.551; 0.663]
Yao-Shuang Li (2024)	204	524	3.1%	2.7%	0.389 [0.347; 0.433]
Minli Liu (2024)	125	308	1.8%	2.7%	0.406 [0.350; 0.463]
Xueyan Liu (2024)	451	898	5.3%	2.7%	0.502 [0.469; 0.535]
Hongying Liu (2024)	187	475	2.8%	2.7%	0.394 [0.349; 0.439]
Pracheth Raghuveer (2025)	63	108	0.6%	2.6%	0.583 [0.485; 0.676]
Xueling Zhou (2025)	140	320	1.9%	2.7%	0.438 [0.382; 0.494]
Fatima Khan (2025)	37	99	0.6%	2.6%	0.374 [0.280; 0.477]
**Total (common effect, 95% CI)**		**12707**	**75.1%**	.	**0.369 [0.361; 0.378]**
**Total (random effect, 95% CI)**		.		**79.0%**	**0.453 [0.379; 0.528]**
Heterogeneity: $Tau^2 = 0.0425$; $Chi^2 = 2960.18$, df = 29 (P = 0); $I^2 = 99\%$					
**continent = North America**					
Pilar Lavielle (2015)	38	1712	10.1%	2.7%	0.022 [0.015; 0.030]
**continent = Europe**					
Calum D. Moulton (2016)	176	980	5.8%	2.7%	0.180 [0.155; 0.204]
**continent = Africa**					
Zainab I. Abba (2018)	33	223	1.3%	2.6%	0.148 [0.105; 0.202]
Fatima Zahra Rhmari Tlemçani (2022)	53	108	0.6%	2.6%	0.491 [0.394; 0.588]
Nimona Ashebir (2024)	184	421	2.5%	2.7%	0.437 [0.388; 0.486]
Endalk Getasew Hiruy (2024)	112	406	2.4%	2.7%	0.276 [0.233; 0.322]
**Total (common effect, 95% CI)**		**1158**	**6.8%**	.	**0.323 [0.296; 0.351]**
**Total (random effect, 95% CI)**		.		**10.5%**	**0.328 [0.183; 0.493]**
Heterogeneity: $Tau^2 = 0.0280$; $Chi^2 = 78.92$, df = 3 (P < 0.0001); $I^2 = 96.2\%$					
**continent = South America**					
Agnieszka Bozanic Leal (2022)	19	62	0.4%	2.5%	0.306 [0.197; 0.437]
**Total (common effect, 95% CI)**		**16921**	**100.0%**	.	**0.305 [0.298; 0.312]**
**Total (random effect, 95% CI)**		.		**100.0%**	**0.408 [0.339; 0.479]**
Heterogeneity: $Tau^2 = 0.0485$; $Chi^2 = 4661.45$, df = 37 (P = 0); $I^2 = 99.2\%$					
Test for subgroup differences (common effect): $Chi^2 = 1622.34$, df = 5 (P = 0)					
Test for subgroup differences (random effects): $Chi^2 = 478.00$, df = 5 (P < 0.0001)					

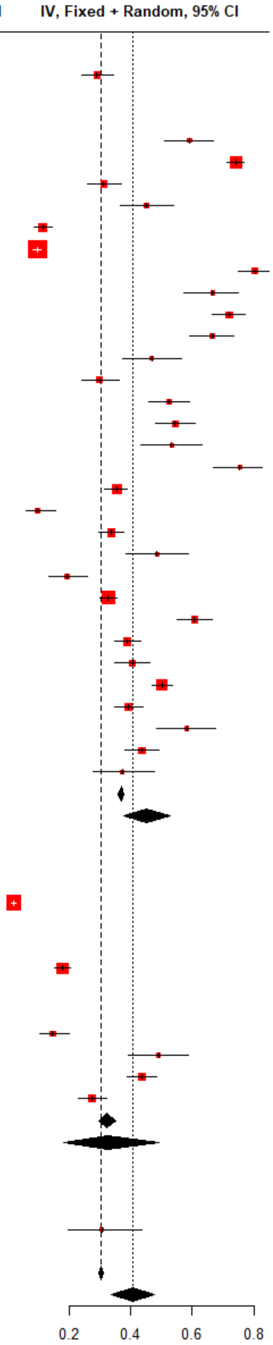

Supplementary Figure S2 Forest plots by continents

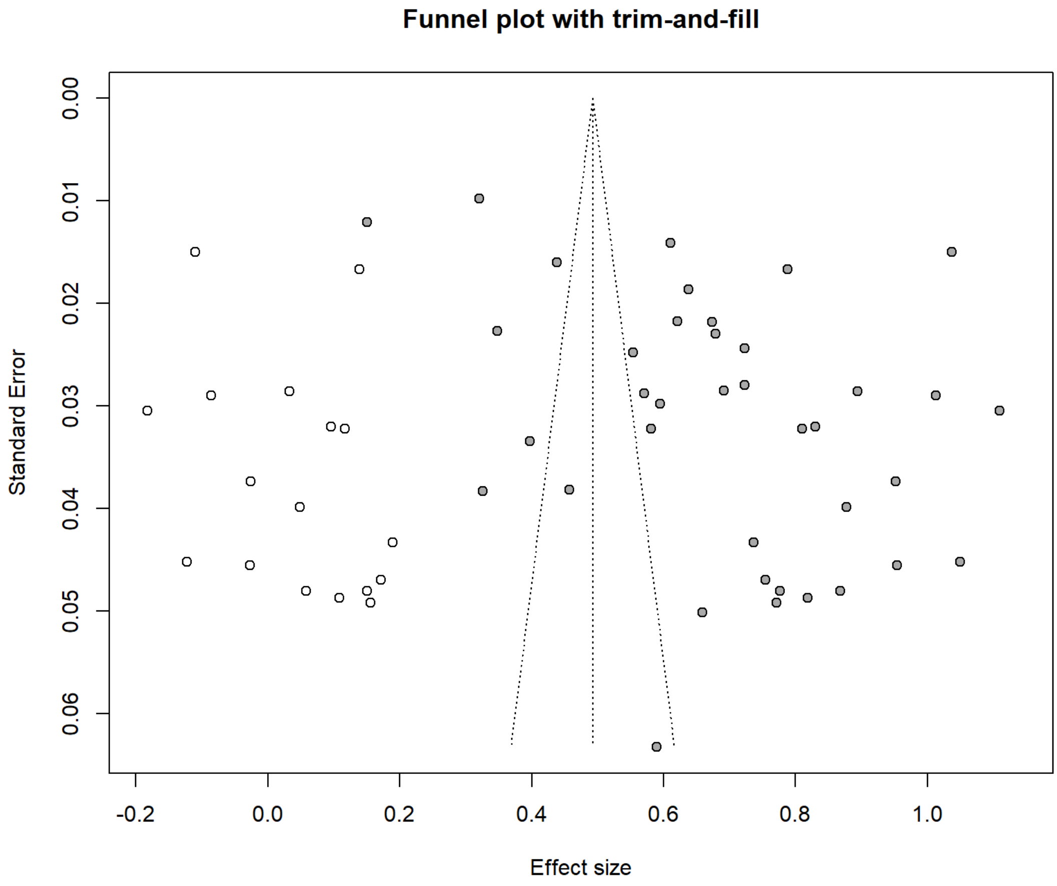

Supplementary Figure S3 Funnel Plot Adjusted Using Trim-and-Fill Metho

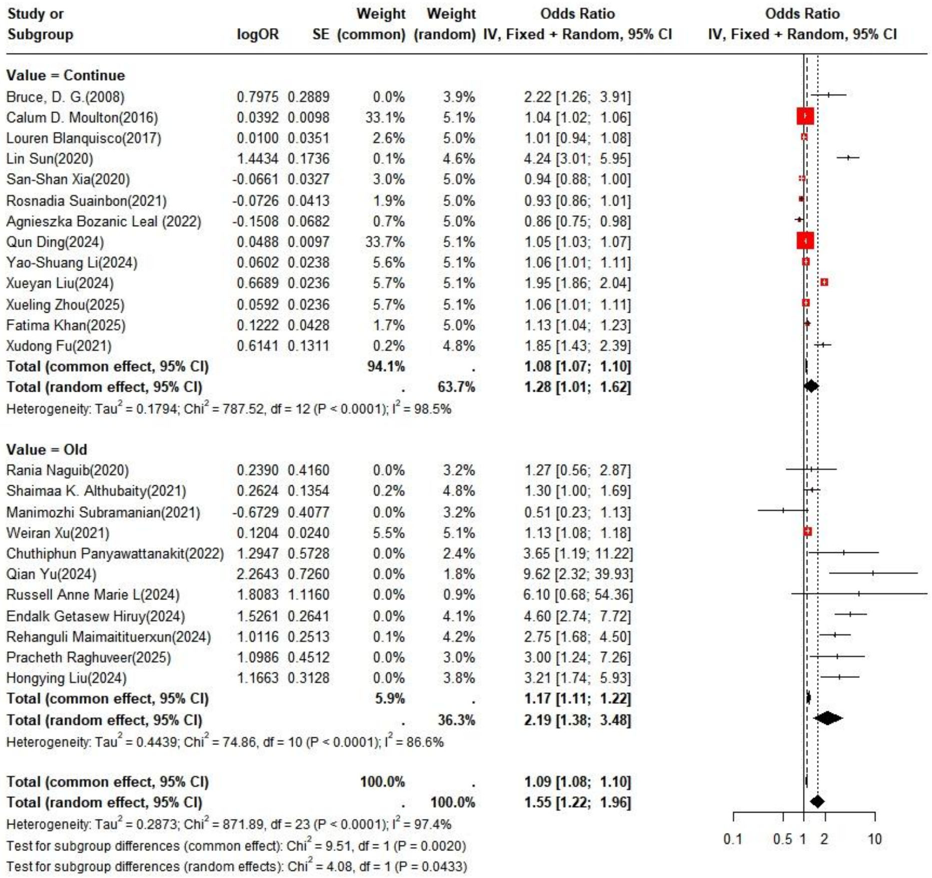

Supplementary Figure S4 Forest plot of age as a risk factor

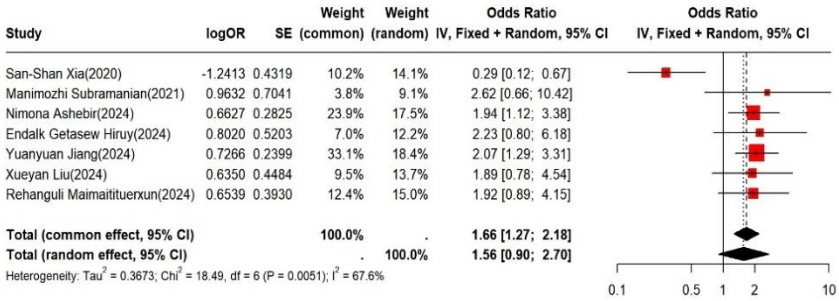

Supplementary Figure S5 Forest plot of Alcohol as a risk factor

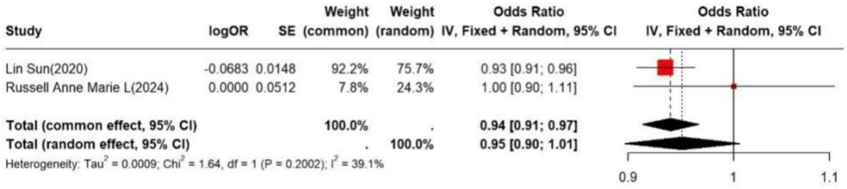

Supplementary Figure S6 Forest plot of BMI as a risk factor

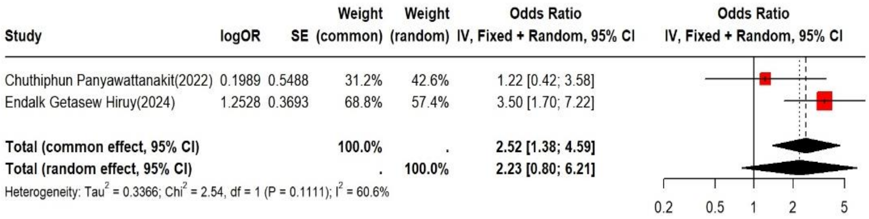

Supplementary Figure S7 Forest plot of Comorbidities as a risk factor

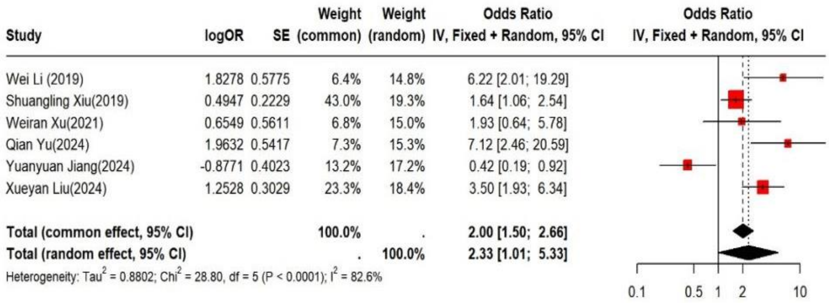

Supplementary Figure S8 Forest plot of Depression as a risk factor

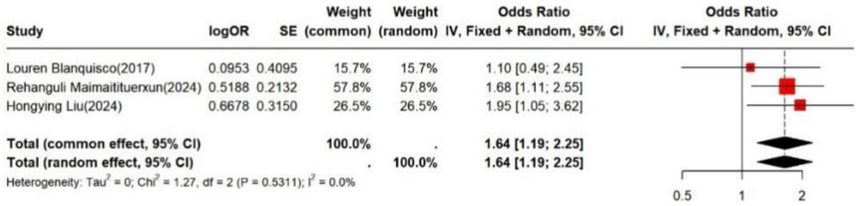

Supplementary Figure S9 Forest plot of Diabetic nephropathy as a risk factor

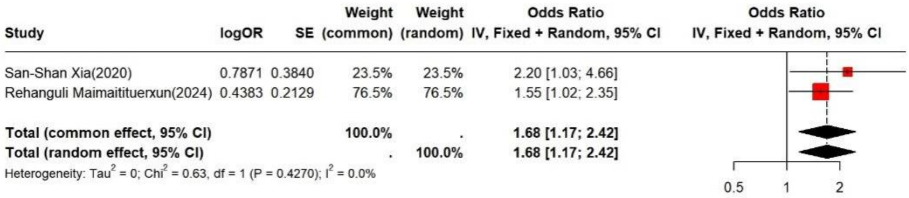

Supplementary Figure S10 Forest plot of Diabetic retinopathy as a risk factor

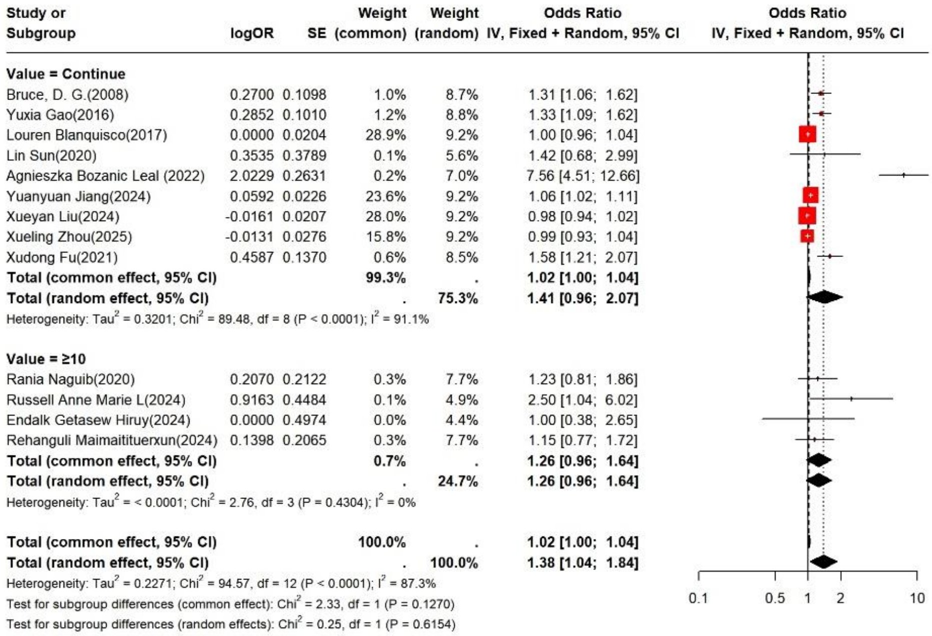

Supplementary Figure S11 Forest plot of Duration diabetes as a risk factor

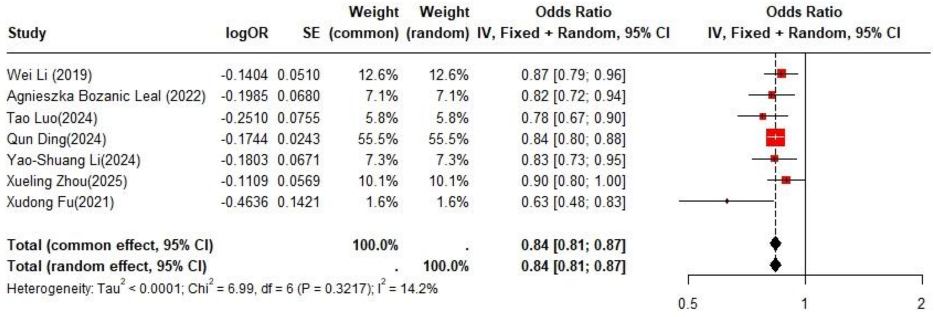

Supplementary Figure S12 Forest plot of Education level as a risk factor

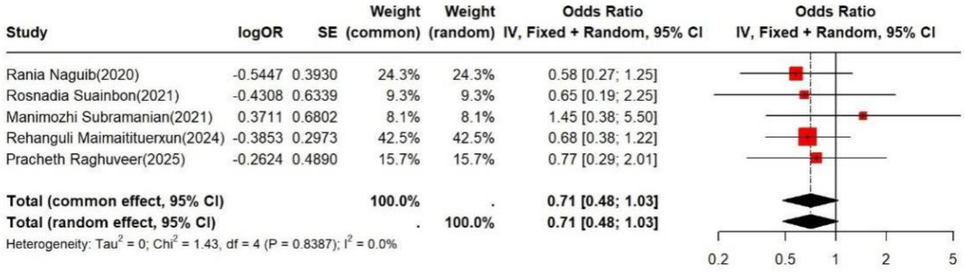

Supplementary Figure S13 Forest plot of Employment as a risk factor

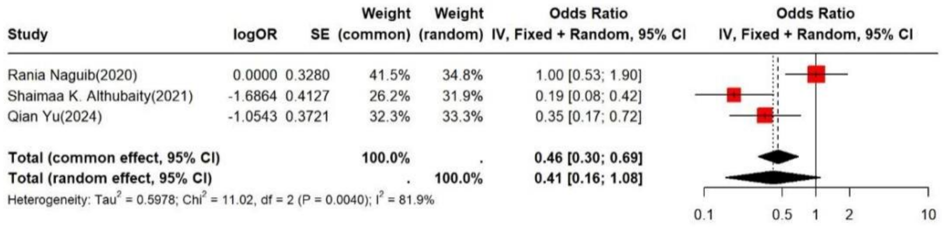

Supplementary Figure S14 Forest plot of Exercise as a risk factor

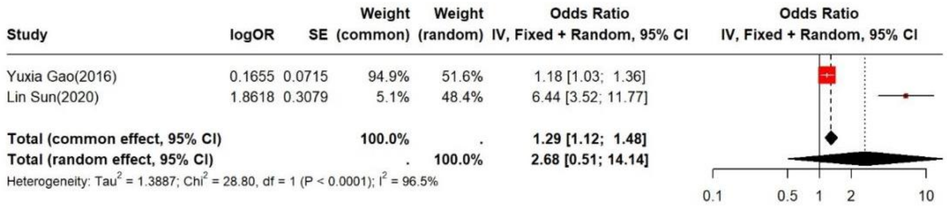

Supplementary Figure S15 Forest plot of FPG as a risk factor

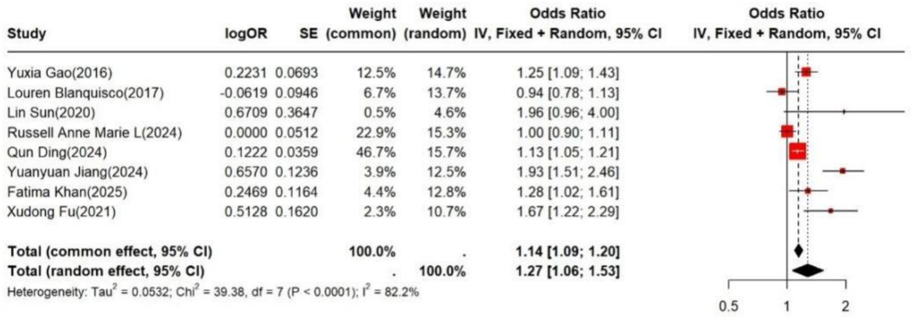

Supplementary Figure S16 Forest plot of HbA1c as a risk factor

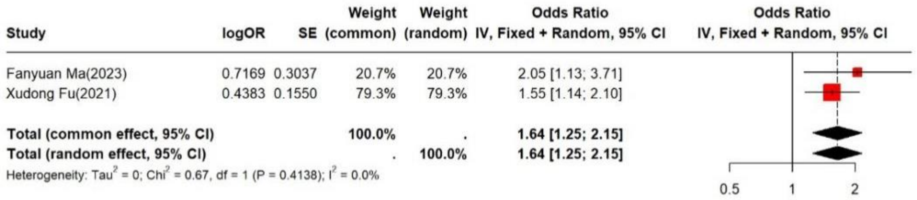

Supplementary Figure S17 Forest plot of HCY as a risk factor

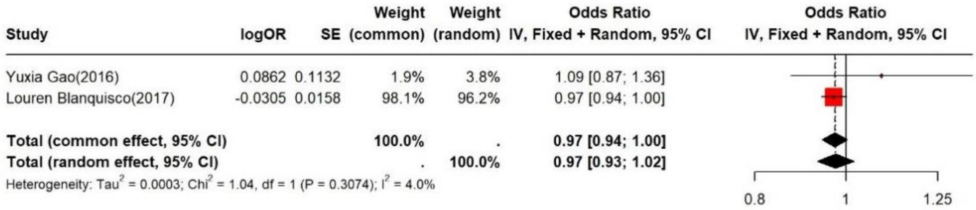

Supplementary Figure S18 Forest plot of HDL as a risk factor

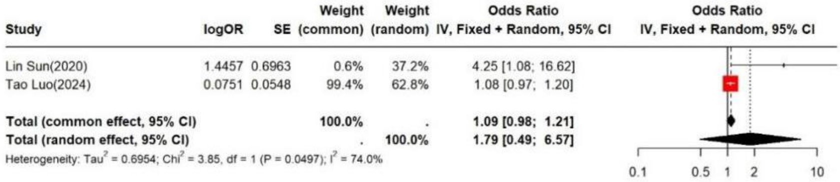

Supplementary Figure S19 Forest plot of HOMA-IR as a risk factor

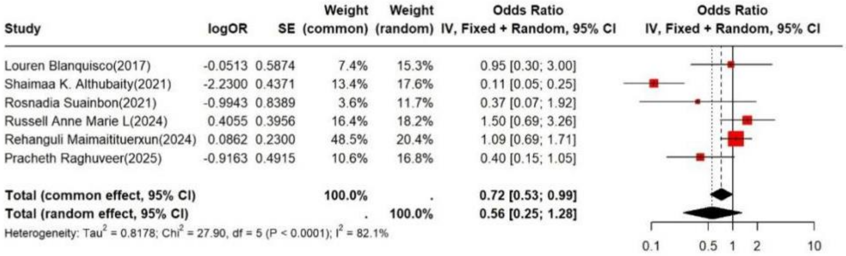

Supplementary Figure S20 Forest plot of Hypertension as a risk factor

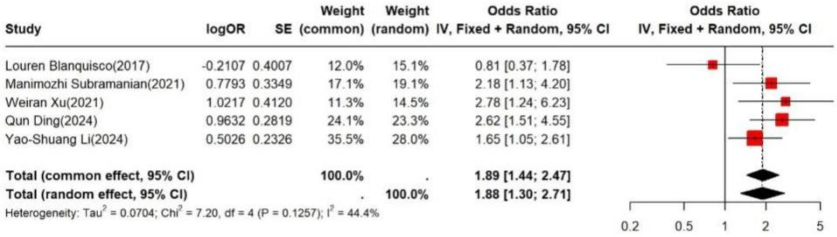

Supplementary Figure S21 Forest plot of Hypoglycemia as a risk factor

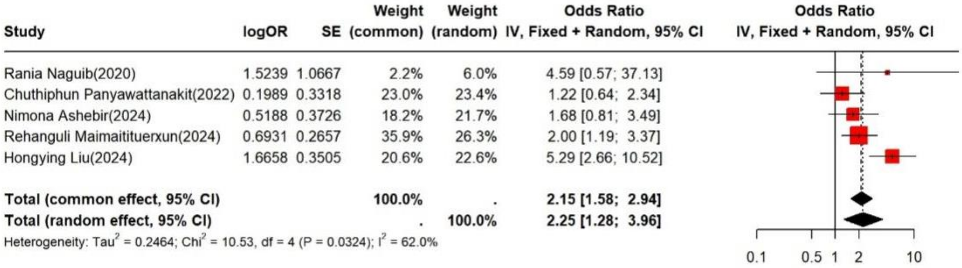

Supplementary Figure S22 Forest plot of Income (Low income) as a risk factor

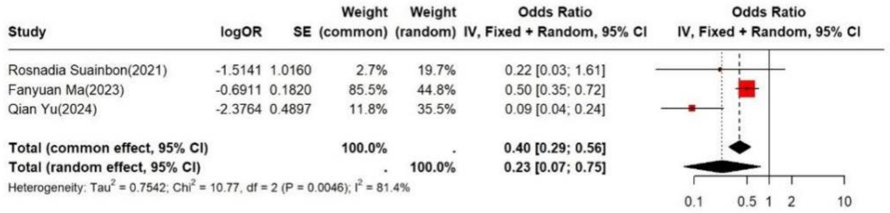

Supplementary Figure S23 Forest plot of Living alone (with Family) as a risk factor

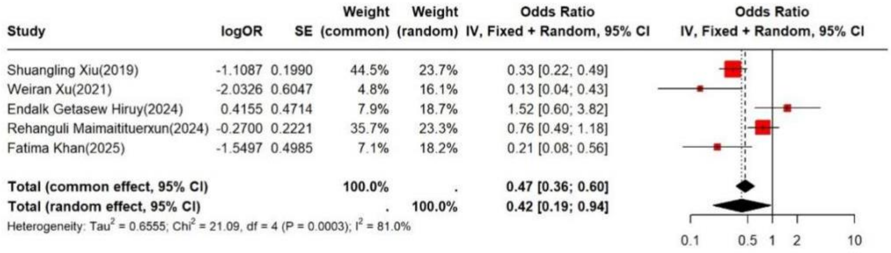

Supplementary Figure S24 Forest plot of Living area (Urban) as a risk factor

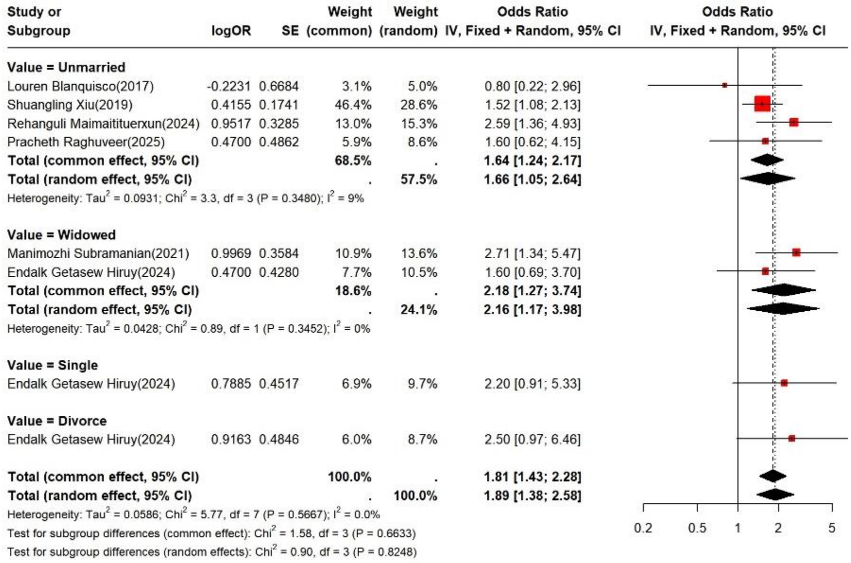

Supplementary Figure S25 Forest plot of Marital status as a risk factor

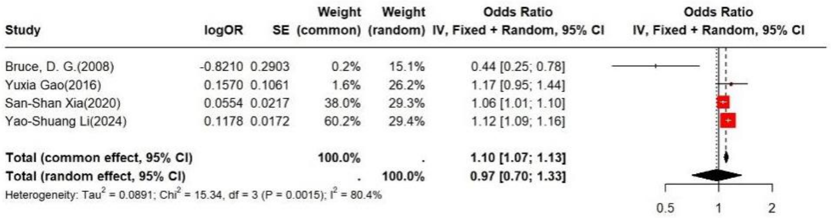

Supplementary Figure S26 Forest plot of Overweight as a risk factor

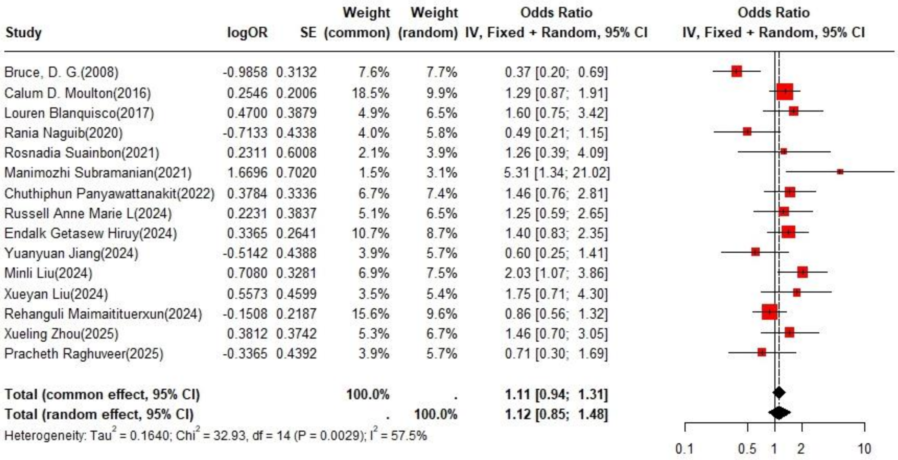

Supplementary Figure S27 Forest plot of Sex (Female) as a risk factor

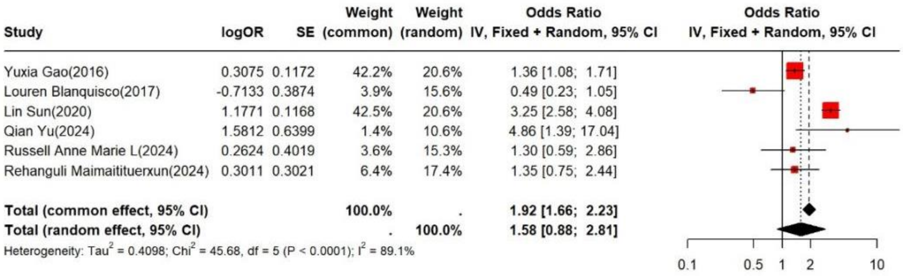

Supplementary Figure S28 Forest plot of Smoking as a risk factor

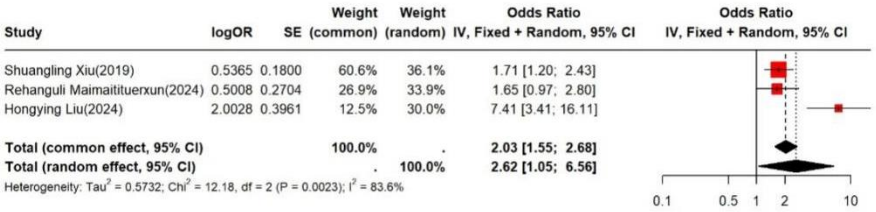

Supplementary Figure S29 Forest plot of Stroke as a risk factor

Study	logOR	SE	Weight (common)	Weight (random)	Odds Ratio IV, Fixed + Random, 95% CI
Yuxia Gao(2016)	0.0677	0.1045	97.3%	97.3%	1.07 [0.87; 1.31]
Lin Sun(2020)	0.2964	0.6312	2.7%	2.7%	1.34 [0.39; 4.63]
**Total (common effect, 95% CI)**			**100.0%**	.	**1.08 [0.88; 1.32]**
**Total (random effect, 95% CI)**			.	**100.0%**	**1.08 [0.88; 1.32]**

Heterogeneity: $Tau^2 = 0$; $Chi^2 = 0.13$, df = 1 (P = 0.7207); $I^2 = 0.0\%$

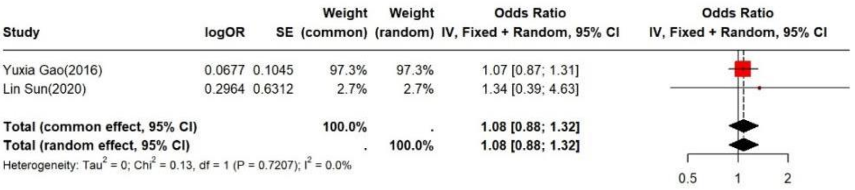

Supplementary Figure S30 Forest plot of TC as a risk factor

References

Abba, Z. I., Mboue-Djieka, Y., Mapoure, Y. N., Nkouonlack, C., Luma, H. N., & Choukem, S.-P. (2018). Prevalence and risk factors of cognitive dysfunction in patients with type 2 diabetes mellitus receiving care in a reference hospital in Cameroon: a cross-sectional study. *International Journal of Diabetes in Developing Countries*, *38*(2), 158-164. https://doi.org/10.1007/s13410-017-0565-2

Althubaity, S. K., Lodhi, F. S., & Khan, A. A. (2021). Frequency and determinants of mild cognitive impairment among diabetic type II patients attending a secondary care hospital in Makkah, Saudi Arabia.

Anand, R., K.Lakshmi, & Srinivasan, A. (2023). A CROSS SECTIONAL STUDY TO ESTIMATE THE INCIDENCE OF COGNITIVE IMPAIRMENT AMONG ADULTS WITH TYPE II DIABETES MELLITUS IN TERTIARY CARE CENTRE IN CHENNAI.

Ashebir, N., Hailesilassie, H., Girma, S., Nigusu, E., & Ezo, E. (2024). Prevalence of Cognitive Impairment and Associated Factors Among Diabetes Mellitus Patients Attending Follow-up Treatment at Fiche General Hospital, North Ethiopia. *SAGE Open Nurs*, *10*, 23779608241227752. https://doi.org/10.1177/23779608241227752

Blanquisco, L., Abejero, J. E., Buno Ii, B., Trajano-Acampado, L., Cenina, A., & Santiago, D. (2017). Factors Associated with Mild Cognitive Impairment among Elderly Filipinos with Type 2 Diabetes Mellitus. *J ASEAN Fed Endocr Soc*, *32*(2), 145-150. https://doi.org/10.15605/jafes.032.02.08

Bozanic, A., Toro, P., Bello-Lepe, S., Hurtado-Oliva, J., Beyle, C., Valdés, C., & Formiga, F. (2022). Cognitive impairment with Type 2 Diabetes Mellitus among community-dwelling older adults in Chile: Prevalence, risk factors and cognitive characteristics. *Front Hum Neurosci*, *16*, 1070611. https://doi.org/10.3389/fnhum.2022.1070611

Bruce, D. G., Davis, W. A., Casey, G. P., Starkstein, S. E., Clarnette, R. M., Foster, J. K.,…Davis, T. M. (2008). Predictors of cognitive impairment and dementia in older people with diabetes. *Diabetologia*, *51*(2), 241-248. https://doi.org/10.1007/s00125-007-0894-7

Carandang, R., Ong, M. T., & Malenab, R. A. J. (2024). Predictors of Cognitive Impairment among Filipino Patients with Type 2 Diabetes Mellitus in a Tertiary Government Hospital. *Acta Med Philipp*, *58*(14), 6-12. https://doi.org/10.47895/amp.vi0.7648

Chen, R. H., Jiang, X. Z., Zhao, X. H., Qin, Y. L., Gu, Z., Gu, P. L.,…Zou, Y. F. (2012). Risk factors of mild cognitive impairment in middle aged patients with type 2 diabetes: a cross-section study. *Ann Endocrinol (Paris)*, *73*(3), 208-212. https://doi.org/10.1016/j.ando.2012.04.009

Ding, Q., Yu, C., Xu, X., Hou, Y., Miao, Y., Yang, S.,…Bi, Y. (2024). Development and Validation of a Risk Score for Mild Cognitive Impairment in Individuals with Type 2 Diabetes in China: A Practical Cognitive Prescreening Tool. *Diabetes Metab Syndr Obes*, *17*, 1171-1182. https://doi.org/10.2147/dmso.S448321

Fu, X., Wang, J., Zhang, P., Du, H., Wu, S., Zhang, H., & Xiong, W. (2021). Diagnosis of TCM symptoms and analysis of risk factors of mild cognitive impairment in patients with type 2 diabetes mellitus. *Am J Transl Res*, *13*(11), 12980-12987.

Gao, Y., Xiao, Y., Miao, R., Zhao, J., Cui, M., Huang, G., & Fei, M. (2016). The prevalence of mild cognitive impairment with type 2 diabetes mellitus among elderly people in China: A cross-sectional study. *Arch Gerontol Geriatr*, *62*, 138-142. https://doi.org/10.1016/j.archger.2015.09.003

Getasew Hiruy, E., Woldegiorgis Abate, T., Animaw Temesgen, W., Bantie, B., Kassaw Yirga, G., Nuru Muhamed, A.,…Walelgn Dessalegn, N. (2024). Cognitive Impairment and Associated Factors Among Adults with Type 2 Diabetes Mellitus in Bahir Dar City Referral Hospitals, 2021: Cross-Sectional Study. *J Alzheimers Dis*, *100*(1), 45-52. https://doi.org/10.3233/jad-230245

Jiang, Y., Liu, X., Gao, H., Yan, J., & Cao, Y. (2024). A new nomogram model for the individualized prediction of mild cognitive impairment in elderly patients with type 2 diabetes mellitus. *Front Endocrinol (Lausanne)*, *15*, 1307837. https://doi.org/10.3389/fendo.2024.1307837

Khan, F., Hussain, S., Singh, S., Sawlani, K. K., Usman, K., Sachan, A. K., & Khattri, S. (2025). A Cross-Sectional Study on the Prevalence and Predictors of Cognitive Impairment and Depression in Elderly Patients With Type 2 Diabetes Mellitus. *Cureus*, *17*(1), e77753. https://doi.org/10.7759/cureus.77753

Lavielle, P., Talavera, J. O., Reynoso, N., González, M., Gómez-Díaz, R. A., Cruz, M.,…Wacher, N. H. (2015). Prevalence of Cognitive Impairment in Recently Diagnosed Type 2 Diabetes Patients: Are Chronic Inflammatory Diseases Responsible for Cognitive Decline? *PLoS One*, *10*(10), e0141325. https://doi.org/10.1371/journal.pone.0141325

Li, W., Sun, L., Li, G., & Xiao, S. (2019). Prevalence, Influence Factors and Cognitive Characteristics of Mild Cognitive Impairment in Type 2 Diabetes Mellitus. *Front Aging Neurosci*, *11*, 180. https://doi.org/10.3389/fnagi.2019.00180

Li, Y. S., Li, J. B., Wang, J. J., Wang, X. H., Jiang, W. R., Qiu, H. N.,…Lin, J. N. (2024). Risk factors for cognitive impairment in middle-aged type 2 diabetic patients: a cross-sectional study. *BMJ Open*, *14*(1), e074753. https://doi.org/10.1136/bmjopen-2023-074753

Liu, H., Feng, Z., Zhang, W., Liu, Y., Xiong, N., Chen, W.,…Dai, W. (2024). Prevalence of cognitive impairment and its associated factors in type 2 diabetes mellitus patients with hypertension in Hunan, China: a cross-sectional study. *Front Psychiatry*, *15*, 1445323. https://doi.org/10.3389/fpsyt.2024.1445323

Liu, M., Wang, Z., Han, J., Mu, Z., & Bian, H. (2024). Analysis of current situation and influencing factors of cognitive dysfunction associated with type 2 diabetes and follow-up study on treatment effectiveness. *Front Neurol*, *15*, 1419017. https://doi.org/10.3389/fneur.2024.1419017

Liu, X., Jiang, T., Jiang, Y., Li, L., & Cao, Y. (2024). Prevalence of mild cognitive impairment and modifiable risk factors: A cross-sectional study in rural older adults with diabetes. *Geriatr Nurs*, *59*, 549-556. https://doi.org/10.1016/j.gerinurse.2024.08.010

Luo, T., Jiang, X., Xu, N., Zhao, X., Xie, X., Xia, X.,…Liu, H. (2024). Risk factors and metabolomics of mild cognitive impairment in type 2 diabetes mellitus. *Front Mol Biosci*, *11*, 1341290. https://doi.org/10.3389/fmolb.2024.1341290

Ma, F., Zhang, Q., Shi, J., Li, S., Wu, L., & Zhang, H. (2023). Risk factors for cognitive dysfunction and glycemic management in older adults with type 2 diabetes mellitus: a retrospective study. *BMC Endocr Disord*, *23*(1), 220. https://doi.org/10.1186/s12902-023-01476-2

Maimaitituerxun, R., Chen, W., Xiang, J., Xie, Y., Xiao, F., Wu, X. Y.,…Dai, W. (2024). Predictive model for identifying mild cognitive impairment in patients with type 2 diabetes mellitus: A CHAID decision tree analysis. *Brain Behav*, *14*(3), e3456. https://doi.org/10.1002/brb3.3456

Moulton, C. D., Stewart, R., Amiel, S. A., Laake, J. P., & Ismail, K. (2016). Factors associated with cognitive impairment in patients with newly diagnosed type 2 diabetes: a cross-sectional study. *Aging Ment Health*, *20*(8), 840-847. https://doi.org/10.1080/13607863.2015.1040723

Murata, Y., Kadoya, Y., Yamada, S., & Sanke, T. (2017). Cognitive impairment in elderly patients with type 2 diabetes mellitus: prevalence and related clinical factors. *Diabetol Int*, *8*(2), 193-198. https://doi.org/10.1007/s13340-016-0292-9

Naguib, R., Soliman, E. S., Neimatallah, F. M., AlKhudhairy, N. S., Alghamdi, A. M., Almosa, R. S.,…Elmorshedy, H. (2020). Cognitive impairment among patients with diabetes in Saudi Arabia: a cross-sectional study. *Middle East Current Psychiatry*, *27*(1), 49. https://doi.org/10.1186/s43045-020-00058-5

Panyawattanakit, C., Wongpradit, W., Kanhasing, R., & Kulalert, P. (2022). Cognitive Impairment and Associated Factors among Older Adults with Diabetes in a Suburban Primary Health Center in Thailand. *Dement Geriatr Cogn Disord*, *51*(2), 175-181. https://doi.org/10.1159/000524132

Raghuveer, P., Shamsudeen, M., Sridhar, S., Singla, R., Gupta, V., Mittal, N.,…Rao, M. (2025). Prevalence and factors associated with cognitive impairment among persons with type 2 diabetes mellitus: A cross-sectional study in medical college hospitals of South India. *Clinical Epidemiology and Global Health*, *31*, 101887. https://doi.org/https://doi.org/10.1016/j.cegh.2024.101887

Rhmari Tlemçani, F. Z., Elamari, S., Motaib, I., Laidi, S., Alidrissi, N., Ahid, S., & Chadli, A. (2022). Factors Associated With Mild Cognitive Impairment in Patients With Type 2 Diabetes: A Cohort Study. *Cureus*, *14*(8), e28305. https://doi.org/10.7759/cureus.28305

Suain Bon, R., Ariaratnam, S., Mat Saher, Z., Mohamad, M., & Lee, F. S. (2021). Cognitive Impairment and Its Associated Risk Factors in the Elderly With Type 2 Diabetes Mellitus. *Front Psychiatry*, *12*, 669725. https://doi.org/10.3389/fpsyt.2021.669725

Subramanian, M., Vasudevan, K., & Rajagopal, A. (2021). Cognitive Impairment Among Older Adults With Diabetes Mellitus in Puducherry: A Community-Based Cross-Sectional Study. *Cureus*, *13*(1), e12488. https://doi.org/10.7759/cureus.12488

Sun, L., Diao, X., Gang, X., Lv, Y., Zhao, X., Yang, S.,…Wang, G. (2020). Risk Factors for Cognitive Impairment in Patients with Type 2 Diabetes. *J Diabetes Res*, *2020*, 4591938. https://doi.org/10.1155/2020/4591938

Xia, S., Zhang, Y., Peng, B., Hu, X., Zhou, L., Chen, C.,…Ji, J. (2022). Detection of mild cognitive impairment in type 2 diabetes mellitus based on machine learning using privileged information. *Neurosci Lett*, *791*, 136908. https://doi.org/10.1016/j.neulet.2022.136908

Xia, S. S., Xia, W. L., Huang, J. J., Zou, H. J., Tao, J., & Yang, Y. (2020). The factors contributing to cognitive dysfunction in type 2 diabetic patients. *Ann Transl Med*, *8*(4), 104. https://doi.org/10.21037/atm.2019.12.113

Xiu, S., Liao, Q., Sun, L., & Chan, P. (2019). Risk factors for cognitive impairment in older people with diabetes: a community-based study. *Ther Adv Endocrinol Metab*, *10*, 2042018819836640. https://doi.org/10.1177/2042018819836640

Xu, W., Hu, X., Zhang, X., Ling, C., Wang, C., & Gao, L. (2021). Cognitive Impairment and Related Factors Among Middle-Aged and Elderly Patients with Type 2 Diabetes from a Bio-Psycho-Social Perspective. *Diabetes Metab Syndr Obes*, *14*, 4361-4369. https://doi.org/10.2147/dmso.S333373

Xu, Z., Zhao, L., Yin, L., Liu, Y., Ren, Y., Yang, G.,…Zhang, G. (2022). MRI-based machine learning model: A potential modality for predicting cognitive dysfunction in patients with type 2 diabetes mellitus. *Front Bioeng Biotechnol*, *10*, 1082794. https://doi.org/10.3389/fbioe.2022.1082794

Yu, Q., Jiang, X., Yan, J., & Yu, H. (2024). Development and validation of a risk prediction model for mild cognitive impairment in elderly patients with type 2 diabetes mellitus. *Geriatr Nurs*, *58*, 119-126. https://doi.org/10.1016/j.gerinurse.2024.05.018

Zhang, X., Xie, J., You, X., & Gong, H. (2023). Risk factors and drug discovery for cognitive impairment in type 2 diabetes mellitus using artificial intelligence interpretation and graph neural networks. *Front Endocrinol (Lausanne)*, *14*, 1213711. https://doi.org/10.3389/fendo.2023.1213711

Zhou, X., Dai, N., Yu, D., Niu, T., & Wang, S. (2025). Development and validation of Galectin-3 and CVAI-based model for predicting cognitive impairment in type 2 diabetes. *J Endocrinol Invest*, *48*(4), 1017-1031. https://doi.org/10.1007/s40618-024-02506-z
